# The Glymphatic System and Meningeal Lymphatics: Current Understandings and Future Perspectives

**DOI:** 10.1002/mco2.70691

**Published:** 2026-03-28

**Authors:** Hangzhe Sun, Haonan Fan, Yuhang Zhou, Haoliang Zhu, Yu Chen, Rui Zhang, Kankai Wang, Yuanbo Pan, Anke Zhang

**Affiliations:** ^1^ The Second Affiliated Hospital School of Medicine Zhejiang University Hangzhou Zhejiang China; ^2^ Wenzhou Medical University Wenzhou Zhejiang China; ^3^ Department of Neurosurgery The Second Affiliated Hospital School of Medicine Zhejiang University Hangzhou Zhejiang China; ^4^ Clinical Medical School Qinghai University Xining Qinghai China; ^5^ Key Laboratory of Precise Treatment and Clinical Translational Research of Neurological Diseases Hangzhou Zhejiang China; ^6^ State Key Laboratory of Transvascular Implantation Devices Hangzhou Zhejiang China

**Keywords:** central nervous system, glymphatic system, meningeal lymphatics, neuroinflammation

## Abstract

The central nervous system (CNS) maintains homeostasis and immune surveillance through a recently defined brain‐wide clearance network: the glymphatic–lymphatic axis. This system couples the intramural glymphatic pathway, responsible for convective fluid transport and parenchymal waste removal, with the meningeal lymphatic vessels (MLVs), which serve as the critical efferent route to the peripheral immune system. This review delineates the structural and functional foundations of each component, their regulatory dynamics, including the roles of sleep and aging, and their synergistic interplay in maintaining fluid balance, clearing metabolic waste, and facilitating neuroimmune communication. Mounting evidence identifies the dysfunction of this integrated axis as a common pathological mechanism across a spectrum of neurological disorders. We highlight its pivotal role in three key paradigms: acute injury (stroke), chronic proteinopathy (Alzheimer's disease, AD), and autoimmune dysregulation (multiple sclerosis, MS), where impaired clearance and maladaptive immune responses are central, recurring themes. The review critically evaluates emerging translational strategies aimed at therapeutically modulating this axis, including pharmacological targets (VEGF‐C, Piezo1 agonists), noninvasive neuromodulation (photo‐biomodulation, PBM), and surgical interventions (lymphaticovenous anastomosis, LVA). This synthesis positions the glymphatic–lymphatic axis as a fundamental physiological network and a pivotal target for novel interventions, outlining key future research directions in neurology.

## Graphical abstract

Brain waste clearance relies on the anatomical and functional integration of the glymphatic system and meningeal lymphatic vessels. Cerebrospinal fluid influx into the brain parenchyma occurs along periarterial spaces, a process driven by polarized aquaporin‐4 (AQP4) water channels on astrocytic endfeet. Solutes are subsequently cleared via perivenous spaces and absorbed by meningeal lymphatics, which drain them to the deep cervical lymph nodes. The efficiency of this pathway is dynamically regulated by factors including sleep, arterial pulsatility, and body posture. Dysfunction of this clearance axis impairs the removal of pathological proteins (amyloid‐β, tau), leading to their accumulation, triggering neuroinflammation, and worsening outcomes after brain injury, thereby playing a key role in neurodegenerative diseases and acute brain injury. Consequently, targeting this pathway to enhance its function represents a promising novel therapeutic direction.

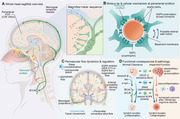

## 1 Introduction

The central nervous system (CNS), once regarded as an immune‐privileged site, faces the ongoing challenge of eliminating metabolic waste while ensuring precise immune surveillance [1, 2]. For over a century, the mechanisms responsible for solute clearance from the brain parenchyma remained poorly understood.

This gap in understanding stemmed from a reliance on passive diffusion models and arachnoid granulation–based drainage, which failed to explain the efficient clearance of proteins such as amyloid‐β [3]. This gap in knowledge impeded our understanding of neurodegenerative disease pathogenesis.

A paradigm shift occurred in 2012, which revolutionized this perspective. The discovery of the glymphatic system uncovered a highly organized, astrocyte‐dependent network that enables convective bulk flow of cerebrospinal fluid (CSF) through perivascular spaces (PVS), providing a mechanism for intracranial waste clearance [4]. Shortly after, the identification of functional meningeal lymphatic vessels (MLVs) in 2015 addressed the next challenge: revealing the long‐sought efferent pathway linking the CNS to the peripheral immune system via the deep cervical lymph nodes (dCLNs) [5, 6]. These two systems are not independent but are intimately connected, forming an integrated glymphatic–lymphatic clearance axis.

This axis is more than a simple “plumbing” system; it dynamically regulates brain homeostasis, fluid dynamics, and neuroimmune communication. Dysfunction in this axis is emerging as a central feature in a diverse range of neurological conditions, from acute stroke and traumatic injury to chronic Alzheimer's disease (AD), Parkinson's disease (PD), and autoimmune disorders such as multiple sclerosis (MS) [7, 8]. Understanding its role provides a unified pathophysiological framework that transcends traditional disease classifications.

This review synthesizes the current understanding of this critical brain clearance axis. It first explores the structural and functional foundations of the glymphatic system and meningeal lymphatics separately and then integrates them into a cohesive physiological circuit, highlighting newly discovered anatomical gateways such as the arachnoid cuff exit (ACE) points. The review examines how dysfunction of this axis manifests in various neurological disorders, using stroke, AD, and MS as case studies to illustrate shared and distinct mechanisms of failure. Finally, it evaluates emerging therapeutic strategies targeting specific nodes of this axis, including pharmacological modulation and surgical interventions, while discussing the challenges ahead in translating these concepts into clinical practice.

## 2 The Glymphatic System: Intracranial Clearance and Fluid Dynamics

The discovery of the glymphatic system has revolutionized our understanding of fluid exchange and waste clearance in the brain. Unlike conventional lymphatic vasculature, it is a specialized network that uses PVS as primary conduits, depends on astrocytic aquaporin‐4 (AQP4) for fluid propulsion, and is critically influenced by the sleep‐wake cycle.

### 2.1 Discovery and Overview of the Glymphatic System

Brain homeostasis is fundamentally reliant on the continuous production and turnover of CSF, with approximately 140 mL present in the adult human brain, primarily produced by the choroid plexus. However, a long‐standing challenge in neurology has been to clarify the mechanisms governing solute clearance from the brain's extracellular space [9]. The accumulation of proteins is a hallmark of AD, PD, amyotrophic lateral sclerosis (ALS), and other neurodegenerative disorders [10, 11, 12, 13]. Similarly, in the aftermath of acute damage such as stroke or traumatic brain injury (TBI), the persistent accumulation of metabolic waste and damage‐associated molecules can exacerbate secondary injury and disease progression [14, 15, 16, 17]. These observations suggest that impaired cerebral solute clearance is a common pathophysiological mechanism underlying both chronic neurodegeneration and poor recovery following acute brain injury.

Before the discovery of the glymphatic system, the dominant understanding of solute clearance from the brain was based on two concepts: the passive diffusion of interstitial fluid (ISF) within the parenchyma and the classic model of CSF drainage via arachnoid granulations into the dural venous sinuses. Although the latter accounted for a major route of CSF volume turnover, it failed to explain the efficient clearance of larger molecules, such as proteins and metabolic wastes [3]. This limitation in the traditional framework led to the search for additional or complementary clearance pathways. Early experimental evidence suggested a lymphatic‐like drainage route from the rodent brain to the cervical lymph nodes, indicating a previously unrecognized component of cerebral clearance [18, 19]. The breakthrough came with advanced in vivo imaging. Using two‐photon microscopy to observe CSF dynamics in live mice, researchers identified and characterized a high‐capacity clearance system, named the “glymphatic” or “glia‐lymphatic” system [4, 20]. This discovery has transformed our understanding of waste removal from the brain and provided a mechanistic explanation for the clearance deficits long implicated in neurological disorders.

### 2.2 Structural Foundation of the Glymphatic System

The glymphatic system is a highly organized fluid transport network whose function relies on the coordinated activity of two key anatomical structures: the PVS and the astrocytic endfeet.

#### 2.2.1 Perivascular Spaces: Structural Conduits for Directed Fluid Flow

PVS are fluid‐filled annular compartments located between the walls of cerebral blood vessels and the surrounding astrocytic endfeet [21]. Structurally, they serve as low‐resistance conduits for fluid transport, with their integrity supported by basement membrane components such as laminin and collagen [4, 6, 20, 22].

PVS within the glymphatic system enable directional bulk fluid flow. This concept evolved through key observations. Early tracer studies indicated that these spaces could facilitate bulk fluid movement but also revealed complex, variable flow patterns [23, 24, 25]. The definitive understanding emerged with advanced in vivo imaging, which demonstrated that CSF influx occurs exclusively along periarterial PVS, whereas ISF and waste are cleared via adjacent perivenous spaces [4]. This model has since become foundational in glymphatic physiology. Recent studies further clarify that macromolecules injected into the CSF migrate from periarterial to perivenous spaces at the arteriovenous overlapping zones within the leptomeninges [26].

Thus, PVS are not passive conduits but are anatomically specialized to establish and maintain the directional fluid dynamics essential for brain‐wide clearance.

#### 2.2.2 Astrocytic Endfeet and AQP4: The Osmotic Engine of Glymphatic Clearance

Astrocytic endfeet form a continuous membranous sheath that envelops cerebral microvessels, representing the essential cellular foundation of the glymphatic system [27]. These specialized structures exhibit a high abundance of the water channel AQP4, primarily localized to perivascular endfeet and the subpial and subependymal glial limiting membranes throughout the CNS [28].

At the molecular level, AQP4 forms tetramers that further assemble into supramolecular clusters known as orthogonal arrays of particles (OAPs) [29, 30]. The specific localization of these OAPs to the perivascular endfeet membrane is mediated by their interaction with the dystrophin‐associated protein complex (DAPC). Within this complex, AQP4 is bound to α‐syntrophin, whereas the DAPC itself is connected to basement membrane proteins, including laminin, via α‐dystroglycan [30]. This specialized anchoring system ensures the concentrated deployment of functional water channels at the perivascular–interstitial interface.

This highly polarized distribution is physiologically critical. AQP4 density at the perivascular endfoot membrane is approximately 40 times higher than at the astrocytic cell body, creating a powerful osmotic sink [31]. This gradient drives the convective influx of water from periarterial spaces into the brain interstitium, generating the directional bulk flow necessary for efficient parenchymal solute clearance. Additionally, this fluid transport is dynamic; optogenetic studies show that pulsatile water movement through AQP4 is temporally coordinated with cerebral arterial pulsations, indicating a mechanism of hemodynamic regulation [32]. The essential role of AQP4 is clearly demonstrated by both genetic ablation and pharmacological inhibition. This is most evident in AQP4‐deficient mice, which exhibit a nearly 70% reduction in interstitial solute clearance rate, and by the suppression of glymphatic fluid transport in the presence of the AQP4 inhibitor AER‐271 [4, 33].

### 2.3 Physiological Regulation and Functions of the Glymphatic System

#### 2.3.1 Core Functions of the Glymphatic System

The glymphatic system plays a pivotal role in maintaining brain homeostasis, with its most defining function being the clearance of metabolic waste, including soluble amyloid‐β and lactate, as evidenced by impaired solute removal in models of glymphatic suppression [4]. Beyond waste disposal, this system facilitates the brain‐wide distribution of nutrients such as glucose and supports the perivascular delivery of therapeutic agents [34]. Additionally, glymphatic flow contributes to volume transmission for intercellular signaling and may play a role in astrocytic mechanotransduction through fluid shear stress [35]. Thus, by efficiently transporting a broad range of molecules, the glymphatic system integrates waste clearance, nutrient supply, drug distribution, and intercellular communication.

#### 2.3.2 Dynamic Regulatory Factors of the Glymphatic System

The function of the glymphatic system is dynamically regulated by several key physiological factors. Natural sleep or anesthesia significantly enhances periarterial CSF influx and interstitial solute clearance, a process attributed to sleep‐associated reductions in noradrenergic tone, which expand the extracellular space and facilitate fluid exchange [36]. Cerebral arterial pulsatility, driven by cardiac, respiratory, and vasomotor cycles, generates essential oscillatory motion within perivascular channels, helping maintain the pressure gradients necessary for net directional CSF flow [37, 38, 39]. Body position also affects clearance efficiency, with the lateral decubitus posture optimizing CSF pathways in animal models [40]. Sleep‐wake state, intrinsic physiological pulsations, and posture collectively modulate the glymphatic system's clearance capacity.

In summary, the glymphatic system is a highly organized network that facilitates CSF‐ISF exchange through perivascular conduits. Its function is regulated by sleep, hemodynamic pulsations, and posture, supporting critical homeostatic processes such as metabolic waste clearance, nutrient distribution, and intercellular signaling. However, a critical gap remained in this clearance model: the glymphatic pathway describes intracranial fluid and solute transport but does not identify the final efflux route from the cranium. This anatomical and physiological missing link set the stage for the rediscovery of the long‐overlooked MLVs.

## 3 MLVs: The CNS Drainage and Immunological Interface

MLVs are crucial conduits for CNS drainage and immune interface. Their definitive identification in 2015 overturned the traditional concept of CNS immune privilege. Primarily located in the dura mater with regional heterogeneity, MLVs facilitate CSF efflux and clearance of metabolic waste. They also serve as channels for immune cell trafficking and antigen presentation, playing a central role in CNS immune surveillance and homeostasis. Their function is vital in neurological disorders, and their existence has been confirmed across multiple species, including humans.

### 3.1 Rediscovery and Paradigm Shift

The long‐standing doctrine of CNS immune privilege established a century‐long paradigm of immunological isolation for the CNS. This view was based on the presumed absence of classical lymphatic drainage and supported by features such as the blood–brain barrier (BBB) and reduced tissue graft rejection [41, 42, 43]. However, anatomical clues challenging this view have existed since the 18th century, from early illustrations to late 20th‐century electron microscopy observations of dural “stomata.” These findings, though intriguing, lacked definitive molecular characterization and were largely overlooked [44, 45].

The paradigm began to shift as questions emerged regarding immune cell trafficking in the meninges [46]. Meanwhile, the discovery of the glymphatic pathway resolved the puzzle of intracranial waste clearance, but it simultaneously raised a new question: where is the exit? A critical breakthrough in understanding CNS fluid dynamics occurred with the description of the glymphatic system in 2012, which outlined an intracranial clearance pathway but left the final efflux route from the cranium unresolved [4]. The definitive turning point came in 2015 when seminal studies led by Kipnis and Aspelund's team independently and conclusively identified a true network of MLVs [5, 6]. Using immunofluorescence staining, they demonstrated that specialized tubular structures along the dural venous sinuses, including the superior sagittal sinus, expressed canonical lymphatic endothelial markers such as LYVE‐1, PROX1, and VEGFR3. This molecular signature definitively established their identity as true lymphatic vessels rather than passive channels.

These studies mapped the anatomical location and functional connection of MLVs: They are primarily located within the dura mater, track alongside major venous sinuses, and form an efferent network that drains directly into dCLNs. This discovery provided the missing physical conduit linking the CNS to the peripheral immune system, resolving longstanding questions about immune surveillance and solute clearance. However, since their rediscovery, subsequent research has continuously refined our understanding of their anatomical distribution. Recent work indicates that MLVs in the human dura mater are not restricted to regions adjacent to the venous sinuses but exhibit a more extensive presence throughout dural areas distant from these sinuses [47].

**FIGURE 1:**
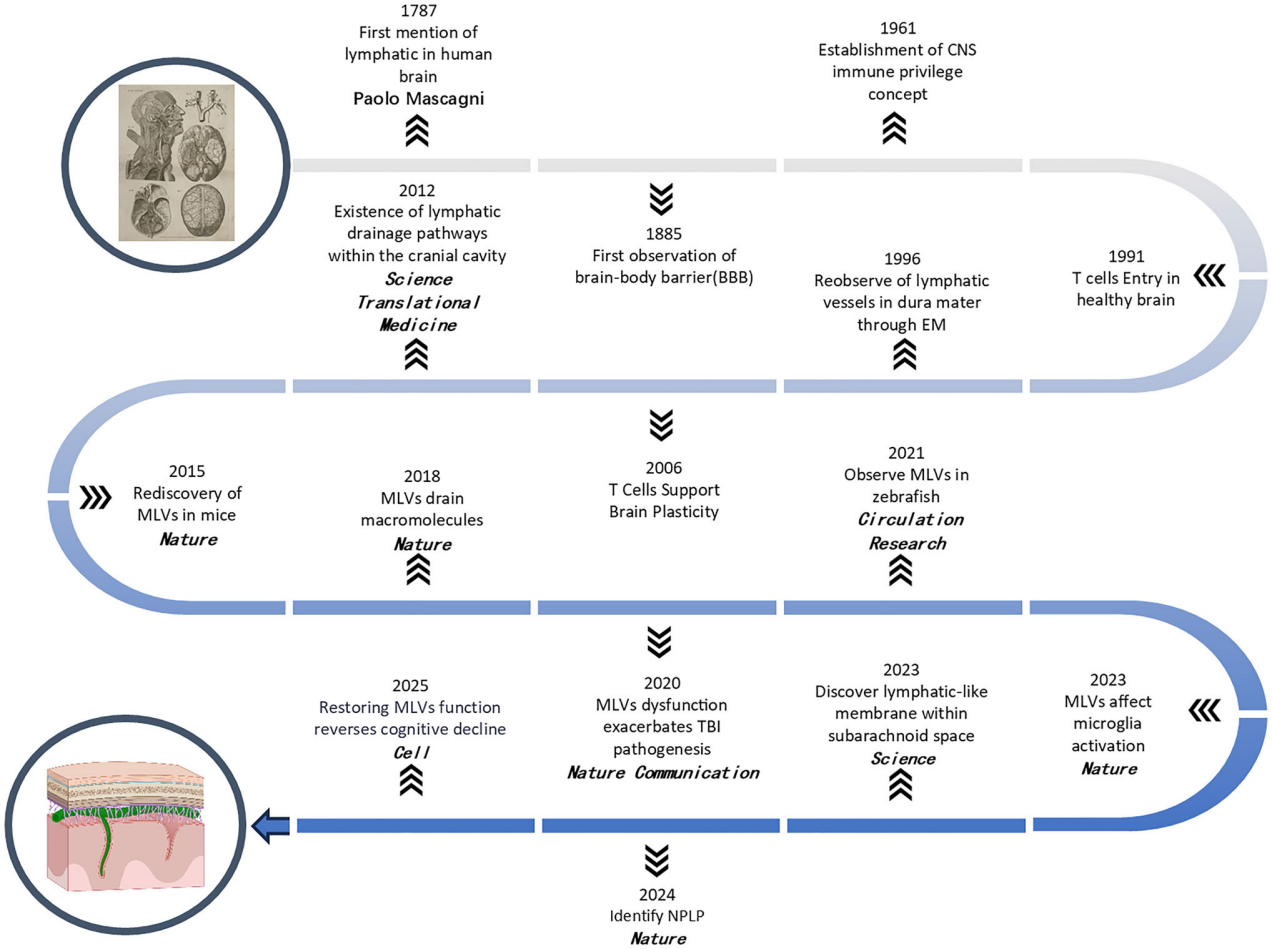
Timeline and key milestones in the discovery of meningeal lymphatic vessels (MLVs). CNS, central nervous system.

Thus, 2015 marked a profound paradigm shift—from viewing the CNS as an immunologically isolated organ to recognizing it as actively monitored by a dedicated meningeal lymphatic system. This re‐discovery has since provided a vital new framework for investigating the pathophysiology of CNS disorders, introducing meningeal lymphatic dysfunction as a novel and pivotal mechanistic component (Figure 1).

### 3.2 Structural Architecture and Regional Heterogeneity

MLVs share key organizational features with peripheral lymphatic systems but exhibit distinct specializations corresponding to their extensive and heterogeneous distribution within the cranial compartment [48, 49, 50]. Their basic structure consists of two main anatomical segments: initial lymphatic capillaries and collecting vessels. Initial capillaries feature discontinuous button‐like junctions, lack valves, and smooth muscle, allowing for high‐permeability drainage. Collecting vessels, in contrast, possess zipper‐like junctions, valves, and smooth muscle, facilitating unidirectional propulsion.

MLVs demonstrate notable regional heterogeneity. The dorsal meningeal lymphatic network, which primarily tracks along the dural venous sinuses, has a sparsely branched architecture with low‐caliber vessels. These dorsal vessels exhibit continuous zipper‐like tight intercellular junctions, suggesting a primary function in the efficient, unidirectional propulsion of pre‐collected lymph. This network converges with its basal counterparts before exiting the skull through foramina, such as those of the emissary veins. In contrast, basal meningeal lymphatics have larger lumina and abundant initial capillary branches with button‐like junctions and valves but lack a smooth muscle layer. These structures specialize in the efficient drainage of CSF and macromolecule exchange [51].

In addition to these two primary networks, a transitional nasopharyngeal lymphatic plexus (NPLP) adjacent to the sinuses has been identified. This plexus displays hybrid structural features between capillaries and collectors, including both junction types, rudimentary valves, and partial smooth muscle investment [52]. NPLP serves as the main route for CSF drainage, suggesting a role in solute filtration and directional fluid transport within complex craniofacial regions [53].

The ethmoidal region, particularly the cribriform plate, is a critical site for CSF efflux [54]. MLVs in this area, termed ethmoidal MLVs, form a distinct network characterized by unique anatomical connections. These vessels establish dorsal linkages with the rostral projections of the superior olfactory sinus and ventral communications with the cavernous sinus region. Notably, ethmoidal MLVs are not directly connected to the dural venous sinuses and do not extend through the ventral or central cribriform plate to access the nasal cavity directly. This specific connectivity suggests a potential role in coordinating or regulating CSF outflow pathways [55]. However, the precise anatomical course of these vessels through the cribriform plate and their definitive functional contribution to drainage processes require further investigation.

Emerging studies have also identified lymphatic or lymphatic‐like structures in other meningeal regions, including leptomeningeal networks and the pial vasculature [56, 57]. A recent study found lymphatic vessels in the mouse brain, specifically in the cortex, thalamus, and hippocampus [58]. However, current evidence supports the consensus that the primary functional pathways for meningeal lymphatic drainage are localized within the dura mater, as emphasized in recent comprehensive reviews [59, 60].

### 3.3 Physiological Functions

#### 3.3.1 Circulation and Efflux of CSF

Prior to the definitive identification of the meningeal lymphatic network, existing models of CSF solute clearance emphasized several distinct efflux pathways. These included direct drainage through arachnoid granulations into the dural venous sinuses and transport along olfactory nerve bundles, traversing the cribriform plate to reach the initial lymphatic vessels of the nasal mucosa [61, 62, 63].

Contemporary research has firmly established MLVs as a major efflux route, accounting for approximately 50% of total CSF drainage in animal models [5, 64, 65]. Within this pathway, subarachnoid CSF enters the brain parenchyma via periarterial spaces and must traverse newly identified ACE points to reach the dural compartment. Subsequently, MLVs absorb this fluid from the dura, facilitating its unidirectional transport to the dCLNs [66]. MLVs adjacent to dural sinuses, including the transverse and sigmoid sinuses, serve as primary conduits, ultimately directing waste to the dCLNs via skull base foramina, such as the intervertebral foramina [6]. The NPLP is thought to function as a regulatory hub in this process [52]. The remaining fraction of CSF efflux (approximately 50%) is handled by spinal routes, with recent evidence highlighting drainage from the spinal compartment to mediastinal, iliac, and sacral lymph nodes, as well as via spinal PVS [67, 68, 69].

#### 3.3.2 Clearance of Metabolic Waste and Cellular Debris

MLVs are a critical pathway for the clearance of metabolic waste from the CNS, with dysfunction directly implicated in the pathogenesis of several neurodegenerative disorders [70, 71]. Beyond metabolic byproducts, this clearance extends to cellular debris. A key function involves the drainage of proteins associated with proteopathies, such as amyloid‐β and tau in AD [72, 73, 74, 75, 76, 77]. Similarly, in PD, MLVs clear pathological α‐synuclein (α‐syn); impaired drainage exacerbates α‐syn deposition, triggers inflammation, and accelerates disease progression [78]. Following acute injuries such as TBI, MLVs also facilitate the removal of cellular debris from the parenchyma to dCLNs, potentially mitigating secondary damage [79, 80]. Fluorescently labeled erythrocytes injected into the CSF were taken up by meningeal lymphatics and drained to CLNs, demonstrating that MLVs can transport blood cells within the CSF [81]. These findings position MLVs not merely as a drainage conduit but as a fundamental component in maintaining CNS metabolic homeostasis.

#### 3.3.3 Maintenance of CNS Immune Homeostasis

Building upon the paradigm shift established by the rediscovery of MLVs, a critical question arises: How do MLVs function not only as conduits but also as active regulators of cerebral immune homeostasis? Far from being passive drainage channels, MLVs serve as a dynamic immunological interface. This role is highlighted by the diverse array of immune cells—macrophages, dendritic cells (DCs), T and B lymphocytes, monocytes, and neutrophils—that populate the meningeal compartment and interact with the unique structural features of MLVs [82, 83, 84, 85]. Through this intricate cellular landscape, MLVs maintain the delicate balance between immune surveillance and neuroinflammation, positioning them as a central hub for brain immune regulation [86].

The initiation of adaptive immune surveillance depends on this pathway. DCs within the CNS, particularly those utilizing the permeable networks of the basal and nasopharyngeal regions, capture antigens such as abnormal proteins and viral debris and then migrate via MLVs to dCLNs for antigen presentation, thus initiating an adaptive immune response [87]. Additionally, tissue‐resident macrophages constitutively express major histocompatibility complex (MHC) molecules, highlighting their immunoregulatory potential to engage in TCR‐mediated interactions and facilitate antigen presentation [88]. This process highlights the role of MLVs as the cornerstone of CNS immune surveillance.

MLVs facilitate selective channels for adaptive immune cell trafficking, demonstrating regional specialization [87]. T cell migration is region‐specific: Central memory T cells patrol CNS borders via MLVs in a CCR7‐CCL21‐dependent manner, facilitated by the expression of leukocyte adhesion molecules on specialized CNS endothelium [89]. The permeable basal MLVs, enriched with chemokine signals, likely serve as the primary site for T cell egress from the CSF compartment. Mechanistically, CCR7‐mediated chemotaxis directs CSF‐derived T lymphocytes and antigen‐presenting cells (APCs) through MLVs toward the dCLNs via NPLP [87]. Similarly, B cells utilize specific MLVs adjacent to dural venous sinuses, such as the transverse and sigmoid sinuses, for migration, enabling access to CNS‐associated compartments or recirculation to peripheral lymphoid organs [90, 91]. This pathway supports their role in humoral immune regulation and may contribute to pathological autoantibody production.

MLVs also play a pivotal role in innate immunity. The clearance of short‐lived neutrophils and their debris following acute injury is a high‐volume task, heavily reliant on the absorptive capacity of the basal MLVs [92]. Additionally, specialized border‐associated macrophages (BAMs) adjacent to MLVs can influence CSF dynamics by modulating arterial pulsatility [93]. This creates a fascinating feedback loop in which immune cells regulate the lymphatic function that drains them, highlighting the complexity of MLVs as an immunologically active niche.

Through mechanisms such as antigen presentation, selective leukocyte trafficking, and innate immune clearance, MLVs form the core of a bidirectional communication hub between the CNS and the peripheral immune system. They not only report CNS status to the periphery but also regulate the access of peripheral immune cells.

In summary, MLVs constitute an essential, multifunctional network vital for CNS health. They facilitate fluid and solute clearance while also serving as a dynamic immunological interface. Regional structural specializations enable this system to perform integrated tasks, from waste removal to adaptive immune cell trafficking. A deeper understanding of MLVs provides critical insights into both basic neurophysiology and the pathogenesis of neurological disorders, opening promising therapeutic avenues.

### 3.4 Cross‐Species Lymphatic Blueprints

Although these findings were primarily derived from mice, meningeal lymphatics have also been documented in other species, including marmosets and human autopsy specimens [94, 95]. For instance, the optical clarity of zebrafish has enabled real‐time imaging of intracranial lymphatic sprouting and immune cell trafficking [95]. In humans and nonhuman primates, high‐resolution magnetic resonance imaging (MRI) has been used to noninvasively describe a meningeal lymphatic network with a three‐dimensional anatomy highly similar to that of rodents [55, 94]. Notably, there are several differences when comparing MLVs in humans and mice. In mice, MLVs are typically found in pairs along the dural sinus, with diameters ranging from 20 to 30 µm [6]. In contrast, human MLVs often form clusters of more than five vessels, with diameters varying widely from 19 to 470 µm [96]. Human MLVs also show gender‐based variation, a difference not observed in mice [97]. Despite these discrepancies, mouse models continue to be valuable for MLV‐related research.

The zebrafish, with its embryos and larvae exhibiting remarkable optical transparency, is an ideal model for in vivo microscopic studies. This transparency enables real‐time, in situ observations of lymphatic vessel development and allows for longitudinal monitoring over several days. Zebrafish also offer significant advantages in genetic manipulation, enabling precise investigation of gene functions through knockout, knock‐in, or editing of target genes. These combined features make zebrafish a superior experimental model for studying the meningeal lymphatic system. For example, meningeal lymphatic endothelial cells (mLECs), first identified in zebrafish, exhibit special capabilities such as phagocytosis, providing a novel direction for investigating the meningeal lymphatic system [98]. In summary, the discovery of an evolutionarily conserved meningeal lymphatic system illuminates a new route for CSF efflux and demonstrates a direct connection between the CNS and peripheral immunity, significantly advancing our understanding of CNS disorder pathophysiology.

## 4 The Glymphatic–Lymphatic Axis: An Integrated Clearance Circuit

The preceding sections have outlined two distinct yet intrinsically connected systems: the glymphatic system, which facilitates the active exchange and clearance of fluids and solutes within the brain parenchyma, and the MLVs, which provide the definitive efferent pathway out of the cranium. It is the functional and anatomical integration of these components that forms the complete glymphatic–lymphatic axis, a sophisticated, brain‐wide clearance circuit essential for maintaining homeostasis. This coupling transforms isolated intracranial processes into a systemic drainage operation, directly linking brain health to peripheral immunity.

The axis operates as a sequential, directional pipeline. The journey begins with the glymphatic influx phase: CSF from the subarachnoid space enters the brain along periarterial spaces, driven by arterial pulsatility and the polarized AQP4 channels on astrocytic endfeet [4]. This influx facilitates the convective exchange of ISF, flushing metabolic wastes such as lactate and pathogenic proteins like amyloid‐β into the perivenous spaces [4].

This cleared fluid, now mixed with CSF, must then access the dural compartment for discharge. Recent research has identified ACE points as critical anatomical gateways at the bridging veins, through which CSF and solutes must traverse from the subarachnoid space into the dural border cell layer and the underlying dural connective tissue [66].

**FIGURE 2:**
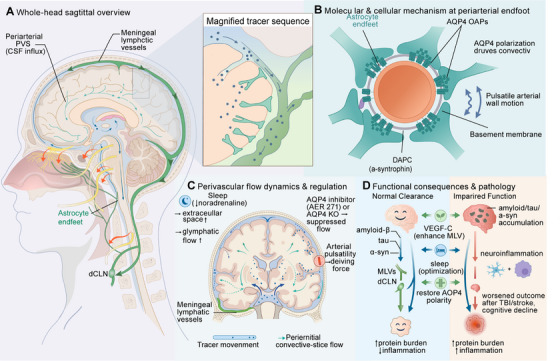
Integration and functional coupling between the glymphatic system and meningeal lymphatic vessels (MLVs). (A) The complete route of cerebrospinal fluid and waste transport from periarterial influx through the interstitium to meningeal lymphatic drainage. (B) The cellular mechanism of glymphatic influx, driven by polarized aquaporin‐4 (AQP4) water channels on astrocyte endfeet. (C) Key physiological and lifestyle factors that regulate the efficiency of the entire clearance axis. (D) Functional consequences, contrasting efficient clearance under normal conditions with pathogenic protein accumulation and neuroinflammation when the system is impaired, alongside potential therapeutic targets. CNS, central nervous system; DAPC, dystrophin‐associated protein complex; dCLN, deep cervical lymph node; OAP, orthogonal arrays of particle; PVS, perivascular spaces; TBI, traumatic brain injury.

The efferent lymphatic phase begins once the fluid reaches the dural compartment. The solute‐rich fluid is primarily absorbed by the basal meningeal lymphatic network. These initial vessels, characterized by button‐like junctions, are specialized for high‐efficiency uptake [51]. The absorbed lymph is then propelled through dorsal collecting lymphatic vessels along the dural sinuses, which feature zipper‐like junctions and intraluminal valves for unidirectional flow [51]. This anatomical sequence ensures that wastes mobilized by the glymphatic system are not recirculated but are instead directed out of the CNS. The circuit concludes with drainage into dCLNs, completing the pathway from the deepest brain parenchyma to the peripheral immune system (Figure 2).

## 5 Dysfunction of the Glymphatic–Lymphatic Axis in Neurological Disorders

Dysfunction of the glymphatic–lymphatic axis emerges as a critical pathological mechanism across neurological disorders. Neuroinflammation commonly triggers this dysfunction, establishing a vicious cycle where impaired waste clearance fuels further inflammation and disease progression. This section analyzes this cycle through three paradigms: the acute collapse and attempted repair in stroke, the chronic, age‐related failure in AD, and the pathogenic conduit role in MS. Understanding this axis provides a unifying framework for diverse conditions and highlights its potential as a therapeutic target.

### 5.1 Neuroinflammation: A Unifying Pathological Framework

**FIGURE 3:**
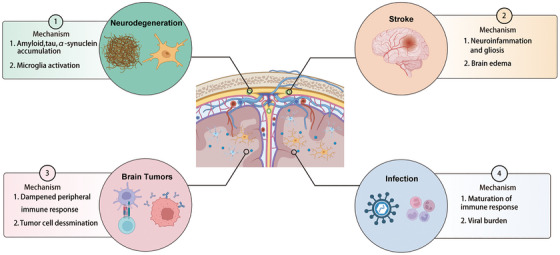
Mechanisms linking meningeal lymphatic vessel (MLV) dysfunction to central nervous system (CNS) diseases. Impaired MLV function contributes to the pathogenesis of major CNS disorders—including neurodegeneration, stroke, brain tumors, and infections—through three principal pathways: impaired clearance and accumulation of brain waste products, activation and perpetuation of neuroinflammation, and facilitation of tumor cell and viral dissemination. Figure created with BioRender.com.

Neuroinflammation, characterized by microglial/astrocytic activation and leukocyte infiltration, represents a universal response to CNS injury and is a hallmark of many neurological disorders, ranging from immune‐mediated MS to acute insults such as stroke and TBI, to chronic neurodegenerative diseases such as AD and PD [99, 100, 101] (Figure 3). Notably, neuroinflammation impairs the function of the glymphatic–lymphatic clearance axis through mechanisms such as pro‐inflammatory cytokine release, BBB disruption, and altered intracranial pressure (ICP). This impairment leads to the accumulation of pathological proteins and disrupts immune homeostasis, which, in turn, fuels further neuroinflammation. Thus, a self‐amplifying vicious cycle is established: Primary neuroinflammation causes clearance axis dysfunction, which exacerbates neuroinflammation, accelerating neuronal damage and disease progression. This common pathological cycle offers a unifying framework for examining a diverse range of neurological conditions.

### 5.2 Acute Insult: The Case of Stroke

#### 5.2.1 Trigger: The Storm of Poststroke Neuroinflammation

Stroke triggers a rapid and profound neuroinflammatory response, albeit with distinct initiators between subtypes: ischemic stroke is driven by hypoxia and danger signals, whereas hemorrhagic stroke is initiated by blood components [102, 103]. This inflammation is amplified by the complement system, creating a vicious cycle of tissue damage [104, 105, 106, 107].

The innate CNS response involves rapid activation of microglia and astrocytes. In ischemia, microglia are activated by hypoxia, DAMPs, and complement factors such as C1q, C3a, and C5a, leading to NF‐κB‐mediated pro‐inflammatory cytokine release (TNF‐α, IL‐1β) [108, 109, 110, 111, 112, 113, 114, 115]. However, in hemorrhage, complement activation is linked to significant tissue damage, with the formation of the membrane attack complex (MAC), which disrupts cell membranes and induces cell lysis [116]. Astrocytes also undergo reactive transformation in both ischemic and hemorrhagic strokes, upregulating markers such as GFAP and vimentin and secreting pro‐inflammatory factors [109, 117, 118]. The triggers for these responses differ: Ischemia involves EAAT2 dysfunction, leading to impaired glutamate clearance and excitotoxicity, whereas hemorrhage is driven by exposure to blood components, promoting a distinct inflammatory phenotype, which plays a central role in proteolytic barrier disruption [119, 120]. Boundary‐associated macrophages (BAMs), particularly perivascular macrophages, contribute to ischemic stroke pathology throughout its course, from early immune responses to the late chronic phase [121]. Following ischemia, subsets such as CD163^+^ BAMs undergo a functional shift toward a pro‐inflammatory state, enhancing leukocyte recruitment and increasing BBB permeability through the induction of vascular endothelial growth factor (VEGF) [122, 123].

Peripheral immune cell infiltration intensifies the inflammatory milieu. In ischemic stroke, neutrophils and macrophages exacerbate secondary injury by worsening vascular damage through the release of reactive oxygen species (ROS) and proteases, such as MMPs [109, 117, 124, 125, 126, 127, 128]. Lymphocytes also play a central role in neuroinflammation. T lymphocytes infiltrate the parenchyma within 24 h after ischemic stroke, peak around Day 3, and remain detectable for weeks [129, 130]. They play complex roles in aggravating or mitigating damage [131, 132]. B lymphocytes also exhibit heterogeneous roles. Guided by ischemic‐induced endothelial upregulation of CXCL13, B cells migrate not only to infarct areas but also to regions supporting functional recovery [133].

In summary, stroke triggers a rapid and multifaceted immune cascade, with innate and adaptive responses collectively disrupting CNS homeostasis. This intense neuroinflammatory environment directly impairs the function of MLVs, the critical hub for fluid and immune clearance, initiating a vicious cycle of clearance failure, as described next.

#### 5.2.2 Collapse: The Breakdown of the Glymphatic–Lymphatic Axis

Stroke‐induced disruption of the BBB and elevated ICP during stroke compromise the integrity of MLVs. The functional consequences, however, diverge between stroke subtypes, reflecting distinct primary injuries.

In hemorrhagic stroke, specific molecular changes, such as the upregulation of THBS1 and S100A6, contribute to MLV dysfunction [134]. This impairment has several significant pathological consequences. First, impaired MLV function critically disrupts the clearance of neurotoxic waste, especially blood breakdown products [135, 136]. This accumulation may establish a pathological link between acute hemorrhagic stroke and long‐term neurodegenerative processes. Interestingly, an early but transient increase in meningeal lymphatic drainage has been observed in a mouse model of intracerebral hemorrhage (ICH), where MLVs dilated 3‐ to 4‐fold within 1 h, leading to rapid hemosiderin accumulation in dCLNs, as well as SAH model [135, 137].

Second, MLV dysfunction disrupts immune cell trafficking, particularly for pro‐inflammatory Th17 cells [138]. In SAH models, altered gene expression in mLECs significantly reduces Th17 cell migratory ability [139]. This failure in immune cell clearance results in the accumulation of pro‐inflammatory cytokines like IL‐17 at the injury site, further perpetuating the inflammatory response [140].

Third, MLV dysfunction can induce maladaptive changes in glial cell gene expression, such as reducing oligodendrocyte numbers and causing lipid metabolism abnormalities. This disrupts myelin stability and promotes demyelination‐related inflammation [141].

In contrast, in ischemic stroke, MLVs facilitate the drainage of injury‐induced VEGF‐C from the CSF to superficial CLNs, leading to the activation of peripheral immune response and, in turn, aggravating the brain injury [142].

#### 5.2.3 Paradox and Struggle: Compensatory Attempts and Dysregulation

Following the initial collapse of the clearance axis, the system mounts a reparative response primarily through lymphangiogenesis—the inflammation‐driven proliferation and remodeling of MLVs [143]. This process represents a critical attempt to restore fluid and immune homeostasis.

The structural compromise of the BBB initiates vasogenic edema, elevating ICP [144, 145]. Elevated ICP not only reflects this failure but also actively exacerbates it by impairing MLV function and glymphatic circulation, creating a vicious cycle [146]. Recent insights suggest that acute postischemic edema may result more from CSF redistribution than from pure vascular leakage, further implicating dysregulation of the clearance pathway [147]. It is within this context of sustained fluid dysregulation that lymphangiogenesis occurs.

Lymphangiogenesis is primarily driven by the VEGF‐C/VEGFR3 signaling axis, involving mediators such as VEGFR2, tyrosine kinase with immunoglobulin‐like and epidermal growth factor‐like domains 2, and delta‐like canonical Notch ligand 4 [64, 75, 76, 148]. The manifestation of lymphangiogenesis, however, is highly context‐dependent: Lymphangiogenesis near the sagittal sinus was observed 2 weeks after photothrombotic ischemic stroke, likely due to direct lymphatic damage. In contrast, transient MCAO showed no evidence of lymphangiogenesis [149]. In ICH, robust lymphatic expansion is observed at later stages, with new MLV branches and increased surface area visible by Day 14, enhancing tracer clearance—particularly in dorsal MLVs—for at least 60 days [135]. This adaptation aids in ISF drainage from injured parenchyma, contributing to edema resolution. In SAH, however, a paradox exists: Improved tracer drainage occurs without detectable lymphatic proliferation, suggesting nonstructural compensatory mechanisms for fluid regulation [81]. After cerebrovascular injury, MLVs may even extend into the damaged brain parenchyma, undergoing lumenization and potentially serving as a scaffold for new blood vessel growth, further promoting tissue repair [149, 150].

In summary, poststroke neuroinflammation triggers a breakdown of the glymphatic–lymphatic axis. Impaired by BBB disruption and elevated ICP, this dysfunction critically hinders the clearance of toxic waste and inflammatory cells, perpetuating a vicious cycle of neuroinflammation. The system attempts compensation primarily through VEGF‐C/VEGFR3‐driven meningeal lymphangiogenesis. However, this reparative response is double‐edged: Although it promotes fluid drainage and structural repair, it may also enhance antigen delivery to peripheral lymph nodes, exacerbating detrimental immune responses.

### 5.3 Chronic Neurodegeneration: The Case of ADs

In stark contrast to the acute failure observed in stroke, AD exemplifies the pathological culmination of a chronic, insidious collapse of the glymphatic–lymphatic clearance axis [151]. Its pathogenesis is marked by a self‐perpetuating cycle, in which age‐related declines in clearance capacity and the accumulation of pathogenic proteins reinforce one another.

This detrimental cycle is initiated by an age‐related baseline failure of the clearance systems. In both rodents and humans, aging undermines critical drivers of waste removal: Arterial pulsatility diminishes, polarized AQP4 water channels are lost at astrocytic endfeet, and MLVs undergo structural retraction [51, 64, 76, 152]. Neuroimaging confirms a concomitant reduction in glymphatic and meningeal lymphatic function in the aging human brain [153, 154]. Emerging evidence indicates that age‐related meningeal immunosenescence is a key driver of this lymphatic decline. Single‐cell RNA sequencing of mLECs in aged mice reveals their heightened response to interferon‐gamma (IFNγ), a cytokine elevated in the aged meninges due to T cell accumulation. Mimicking chronic IFNγ exposure in young mice through AAV‐mediated overexpression recapitulates the impaired CSF drainage observed in aging, providing a direct mechanistic link between immune aging and lymphatic dysfunction [155]. This functional decline is not benign; experimental disruption of either glymphatic (via AQP4 knockout) or meningeal lymphatic function in rodents directly induces deficits in spatial learning and memory, confirming its causal role in cognitive impairment [156].

In AD, this preexisting vulnerability escalates into a definitive pathology. Postmortem analyses reveal a specific glymphatic defect in the AD brain: A marked loss of polarized AQP4 despite increased overall immunoreactivity, indicating a failure in the fluid influx mechanism essential for clearance [156, 157]. The clinical relevance of this defect is highlighted by a strong inverse correlation between the severity of cerebral Aβ angiopathy and perivascular AQP4 levels in patients, highlighting the connection between impaired glymphatic function and toxic protein accumulation. Notably, the observation that overt structural atrophy of MLVs is not a consistent early feature in AD suggests that the initial pathology is primarily functional and physiological [96, 158]. This is further supported by interventional evidence; impairing meningeal lymphatic function alone is sufficient to exacerbate Aβ pathology and cognitive decline in AD models, confirming the axis's active role in disease progression.

Thus, in AD, the glymphatic–lymphatic axis plays a central role in a slow‐motion catastrophe. Its age‐related decline—driven by both structural changes and immune‐mediated functional suppression—creates a vulnerable substrate. The subsequent functional disintegration, primarily through loss of AQP4 polarization and impaired drainage, fosters a permissive environment for Aβ accumulation. This accumulation further disrupts the cerebral milieu, reinforcing the clearance failure and locking the system into a vicious cycle that drives progressive neurodegeneration.

### 5.4 Autoimmune Dysregulation: The Case of MS

MS is a debilitating neuroinflammatory and autoimmune disorder characterized by demyelinating lesions and significant immune cell infiltration into the CNS [159]. As a critical interface between the CNS and the peripheral immune system, MLVs are poised to influence disease pathogenesis by draining tissue antigens and APCs to dCLNs.

Rather than merely mediating immune surveillance, MLVs become a major conduit for the pathogenesis of experimental autoimmune encephalomyelitis (EAE), a model for MS. Impairing meningeal lymphatic drainage—through either local photodynamic ablation or ligation of dCLN afferents—delays disease onset and reduces EAE severity [87]. Interventions targeting nasal lymphatics or brachial lymph nodes do not produce the same effect, emphasizing the specific importance of the MLV‐dCLN axis [87]. The dCLNs serve as a critical site where CNS‐derived antigens and APCs may activate encephalitogenic T cells, as evidenced by the detection of brain‐derived antigens in the dCLNs of both patients with MS and EAE models [87, 160, 161]. However, surgical excision of the dCLNs, while delaying disease progression, does not fully prevent EAE, suggesting the activation of alternative drainage pathways when the primary route is compromised [87, 162, 163].

Emerging evidence also suggests that MLVs may regulate the supply of myeloid cells to the inflamed CNS by controlling CSF influx into the adjacent skull bone marrow [164, 165]. Therefore, understanding the precise interactions between immune cells and MLVs, as well as the immunological events within the dCLNs, holds significant therapeutic relevance for MS.

Interestingly, although VEGFC expression is upregulated in the CNS of rodents with EAE, meningeal and spinal cord lymphatic structures do not exhibit significant morphological expansion [166, 167]. In contrast, lymphatic vessels in the nasal cavity show notable expansion during later or severe stages of EAE, likely through the proliferation of existing vessels [87, 166]. This differential response may be due to intrinsic heterogeneity between lymphatic beds, variations in local growth factor availability, or may indicate that meningeal lymphatics undergo primarily functional or transcriptional alterations during neuroinflammation [168].

In summary, dysfunction of the glymphatic–lymphatic axis represents a core pathophysiological mechanism underlying a broad spectrum of CNS disorders. This review has highlighted its critical role in acute injury (stroke), chronic proteinopathy (AD), and autoimmune dysregulation (MS). However, the influence of this axis extends far beyond these conditions. In brain tumors, altered function may modulate the tumor microenvironment, immune surveillance, and the distribution of chemotherapeutic agents [169, 170, 171, 172]. In migraine, it is implicated in clearing neurotoxic substances following cortical spreading depression and regulating neurogenic inflammation [173]. Following TBI, both acute failure and chronic dysfunction of this system serve as a critical link between the initial insult and long‐term neurodegenerative sequelae [80]. During CNS infections such as meningitis and encephalitis, the axis's role in draining pathogens, inflammatory cells, and cytokines directly impacts infection outcomes and the extent of neurological damage [174, 175, 176]. Consequently, targeting the repair or modulation of this clearance axis not only provides a novel mechanistic framework for understanding these diseases but also represents a promising, cross‐disciplinary therapeutic strategy. Future research must elucidate the disease‐specific regulatory mechanisms of this axis and advance the translation of related therapeutic approaches into clinical practice.

## 6 Therapeutic Strategies Targeting Key Aspects of Glymphatic–Lymphatic Dysfunction

**FIGURE 4:**
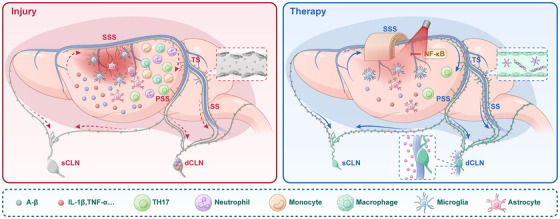
Meningeal lymphatic dysfunction exacerbates stroke injury, whereas therapeutic strategies restore fluid drainage and mitigate neuroinflammation. (A)Injury disrupts MLVs, impairing CSF drainage and waste clearance, elevating ICP, and promoting the migration of immune cells that amplify neuroinflammation. (B) Therapeutic strategies such as VEGF‐C delivery and lymphaticovenous anastomosis restore MLV function and CSF drainage, enhancing the clearance of waste and inflammatory mediators while reducing parenchymal immune cell infiltration. CSF, cerebrospinal fluid; dCLN, deep cervical lymph node; ICP, intracranial pressure; MLVs, meningeal lymphatic vessels.

Emerging evidence positions MLVs as a potential therapeutic target for CNS disorders, with several innovative strategies currently under investigation (Tables 1 and 2, Figure 4).

**TABLE 1:** The functions of meningeal lymphatic vessels (MLVs) in central nervous system (CNS)‐associated disease.

Functions of MLVs	Related diseases/Context	Animal model	Age in weeks	Mechanism/Pathway	Outcomes/Key findings	Experiment intervention/Approach	Potential treatment method	Author	Year	Reference
Metabolic waste clearance	Alzheimer's disease (AD)	5xFAD	20–24 months	Aβ clearance via CSF drainage	MLVs dysfunction occurs in early AD stages	① Ablation of MLVs ② Delivery VEGF‐C through AAV1‐CMV‐mVEGF‐C	① VEGF‐C delivery ② LVA (controversial)	Da Mesquita et al.	2018	[76]
		5xFAD APP/PS1	6 and 11 months, respectively	① Enhance lymphatic drainage ② Reduce Aβ deposition	① Improve cognition ② Restore meningeal lymphatic endothelial cell function	Near‐infrared light	Noninvasive light therapy	Wang et al.	2024	[177]
	Parkinson's disease (PD)	A53T	18 weeks	① α‐synuclein clearance ② Enhance CSF drainage	① α‐synuclein aggregate ② Gliosis, proinflammatory cytokine expression ③ Tau aggregate	ligation of dCLNs afferents	LVA (controversial)	Zou et al.	2019	[178]
Immune cell trafficking	Brain tumors (glioma)	C57BL/6	—	① VEGF‐C/CCL21‐CCR7 axis ② Enhance immunotherapy	Dorsal MLVs remodel to drain tumor antigens to dCLNs	Inject glioma cells engineered to overexpress VEGF‐C or B16 melanoma cells	① VEGF‐C delivery ② Anti‐PD‐1/CTLA‐4 therapy	Luo et al.	2020	[169]
		C57BL/6	8 weeks	Enhance lymphatic drainage	① Increase dendritic cell trafficking ② Increase CD8+ T cell activation ③ Enhance checkpoint therapy responses	Use checkpoint inhibitor	Combine with Immunotherapy	Song et al.	2020	[170]
	Multiple sclerosis (MS)	C57Bl/6J	—	① VEGF‐C signaling ② Connect MLVs to the dCLNs	① Delay the onset and attenuated the severity of EAE ② Alter disease progression ③ Influence T cells accumulation in the dCLNs	① Photodynamic ablation of nasal lymphatics ② Ligation of afferents to the brachial lymph nodes	① VEGF‐C delivery ② Target signaling pathway	Louveau et al.	2018	[87]
Neuroinflammation modulation	AD	5xFAD male	Adult	① IL‐6 signaling ② VEGF‐C/VEGFR3 signaling	CBM surgery reduces neuroinflammation	Cranial bone maneuver (CBM) surgery	Cranial bone maneuver (CBM) surgery	Lu et al.	2025	[179]
		Mice	7–8 months	Improve the clearance of toxic proteins	① Reduce neuroinflammation ② Increase Polarized AQP4 ③ Improve CSF influx ④ Increase Synaptic proteins	Voluntary wheel running (6 weeks)	Exercise	He et al.	2017	[180]
		APP/PS1	5 months	Improve the clearance of toxic proteins	① Reduce neuroinflammation ② Increase expression of Polarized AQP4 ③ Improve CSF influx	Voluntary wheel running (8 weeks)		Liu et al.	2022	[181]
	Stroke	C57BL/6J Vegfr3::YFP	—	VEGF‐C/VEGFR3 signaling activation	Stimulate lymphatic vessel growth and function	Inject VEGF‐C protein	VEGF‐C Delivery	Boisserand et al.	2024	[182]
Erythrocyte clearance	Subarachnoid hemorrhage (SAH)	C57BL/6 male SAH model	6–8 weeks	Enhance lymphatic drainage	Transport red blood cells to CLNs	VEGFR3 blockade	VEGF‐C Delivery	Wang et al.	2020	[81]

Abbreviations: AQP4, aquaporin‐4; CSF, cerebrospinal fluid; dCLN, deep cervical lymph nodes; LVA, lymphaticovenous anastomosis.

**TABLE 2:** Summary of therapy strategies targeting meningeal lymphatic vessels (MLVs).

Therapy strategy	Mechanism	Evidence strength	Characteristics	Limitations	Clinical readiness	Technical complexity	Cost	Safety	Patient suitability	References
VEGF‐C Delivery	① Enhance the function of the meningeal lymphatic system ② Promote MLVs formation ③ Improve CSF and ISF drainage ④ Reduce brain edema ⑤ Promote immune cell transport	Medium animal model/Early‐stage clinical trials	Promotes lymphatic vessel formation	① Long‐term effects unclear ② Potential off‐target effects ③ Specific molecular mechanisms need further clarification	Low	Complex	Medium	Medium	Patients with poststroke brain edema and impaired immune function	[75, 76, 169, 182]
Photo‐biomodulation (PBM)	① Stimulate the meningeal lymphatic system ② Enhance lymphatic drainage ③ Waste clearance ② Reduce neuroinflammation	Low animal model/Few clinical cases	Noninvasive	① Optimal parameters (wavelength, intensity, duration) unclear ② Specific mechanisms need further clarification	Low	Simple	Low	High	Patients with concerns about invasive treatment	[177, 183, 184, 185]
Cranial bone transport (CBM)	① Promote angiogenesis and neurogenesis ② Affect the connection between cranial bone and brain blood vessels and lymph	Low animal model/Few clinical cases	Minimally invasive and long‐lasting efficacy	① Specific mechanisms need further clarification ② Lack high‐quality clinical trials	Low	Medium	Medium	Medium	Needs further assessment; currently not recommended for routine use	[179, 186]
Target signaling pathways	Influence the development and function of MLVs	Medium animal model/Early‐stage clinical trials	High efficiency	① Complex signaling pathways ② Need further research to identify potential therapeutic targets	Low	Complex	High	Low	Needs genetic testing; for patients with specific signaling pathway abnormalities	[187, 188]
Surgical techniques and anastomosis	Improve lymphatic drainage	Low animal models/Clinical case reports	Target MLVs directly	① Still in development ② Needs further refinement and verification ③ Potential surgical risks	Low	Complex	High	Low	Neurodegenerative disease patients; stroke patients need cautious assessment	[189]

Abbreviations: CSF, cerebrospinal fluid; ISF, interstitial fluid; MLVs, meningeal lymphatic vessels.

### 6.1 Photo‐Biomodulation (PBM)

PBM is a noninvasive therapeutic technique that uses light at specific wavelengths, typically within the red or near‐infrared spectrum. Due to its superior tissue penetration capacity, near‐infrared light (600–1100 nm) can be applied transcranially to reach intracranial structures, making it a promising candidate for treating CNS disorders [183, 190, 191].

Preclinical studies provide initial support for this potential. In mouse models of AD, transcranial PBM has been shown to improve memory and cognitive performance, reduce Aβ plaque deposition, enhance meningeal lymphatic drainage, and restore LEC function [177]. The proposed underlying mechanisms may involve increased nitric oxide production, improved cellular metabolism, and enhanced lymphatic contractility [184, 185]. Specific near‐infrared wavelengths, such as 1070 nm, have also been reported to promote the phagocytic clearance of Aβ by microglia [192]. Research in models of TBI and stroke suggests that PBM may promote tissue repair, protect neurons, and improve mitochondrial function and local microcirculation [193].

Despite these encouraging findings, significant challenges remain before PBM can be reliably translated into therapy. The field suffers from a lack of standardized treatment parameters, with core protocols for optimal wavelength, irradiance, frequency, and treatment duration largely based on empirical exploration. This compromises the comparability and reproducibility of results across studies. Most critically, current evidence supporting PBM's efficacy is primarily derived from animal models, with high‐quality clinical data from large‐scale trials in specific patient populations, such as stroke survivors, still lacking. Thus, the definitive role of PBM in treating brain disorders will depend on future rigorously designed clinical trials that can clarify its mechanisms, establish its efficacy, and determine its optimal indications and long‐term safety profile.

### 6.2 Piezo1 Agonist

Piezo1 is a critical mechanosensitive ion channel involved in essential physiological processes, including osmotic and blood pressure regulation, as well as vascular and lymphatic development [194, 195, 196]. It plays a vital role in the formation of lymphatic valves and the proliferation of LECs [197, 198]. Dysfunction of Piezo1 is linked to lymphatic disorders, highlighting its importance in maintaining the integrity of the lymphatic system [199].

Emerging research highlights the pivotal role of Piezo1 in the development and function of MLVs. Activation of Piezo1, through genetic overexpression or pharmacological agonists like Yoda1, enhances CSF drainage by improving lymphatic absorption and transport [200]. This mechanism is particularly relevant for treating pathological CSF accumulation, as seen in conditions like hydrocephalus. For example, in mouse models of hydrocephalus or Down syndrome, where MLVs are often underdeveloped, targeted activation of Piezo1 in lymphatic vessels or systemic administration of Yoda1 significantly reduces pathological CSF buildup, ventriculomegaly, and associated symptoms [200]. The therapeutic potential of this pathway extends to other conditions with impaired CSF dynamics. In aged mice, where CSF drainage is reduced and ICP is elevated, Yoda1 treatment has been shown to restore MLV networks, improve lymphatic drainage, and normalize CSF perfusion [201]. These findings align with studies demonstrating that VEGF‐C‐induced lymphangiogenesis can also rescue MLV function and alleviate ICP in craniosynostosis models, suggesting that combinatorial strategies targeting both structural growth (via VEGF‐C) and functional activation (via Piezo1) may be beneficial.

However, critical translational barriers must be overcome. Yoda1 has pleiotropic effects due to widespread Piezo1 expression, posing risks for off‐target actions. Its poor BBB permeability limits central delivery, and the long‐term safety of chronic activation is unknown. Efficacy in genetically defined animal models may not translate to complex human diseases. Future work requires developing selective agonists and establishing safe, effective delivery strategies.

### 6.3 VEGF‐C Delivery

VEGF‐C is a key regulator of lymphangiogenesis, promoting the growth and proliferation of lymphatic vessels. It has shown neuroprotective effects in preclinical models of various CNS disorders. Da Mesquita et al. found that adeno‐associated virus (AAV)‐mediated delivery of VEGF‐C into the CSF and transcranial delivery of hydrogel‐encapsulated VEGF‐C peptide improved clearance ability and increased CSF drainage into the dCLNs in AD model mice [76]. Similarly, Wen et al. injected recombinant VEGF‐C protein and reached similar conclusions [75]. Furthermore, AAV‐mediated delivery of VEGF‐C into the CSF has been shown to expand the meningeal lymphatic network and improve outcomes in murine stroke models [182]. These findings suggest that VEGF‐C therapy holds promise for stroke treatment. However, a recent study revealed that VEGF‐C may play a damaging role acutely while having a pro‐angiogenic effect chronically.

Although preclinical studies confirm that VEGF‐C promotes meningeal lymphangiogenesis and improves waste clearance and neurological function in animal models, its translational application faces several challenges. Potential risks associated with VEGF‐C overexpression, such as increased vascular permeability and inflammation, need careful consideration [202]. The effects of VEGF‐C are highly time‐dependent: During the acute phase of stroke, it may exacerbate vasogenic edema, whereas in the chronic phase, excessive lymphangiogenesis may disrupt immune homeostasis. Moreover, safe and efficient delivery strategies remain a significant bottleneck. The long‐term expression profile and potential immune responses associated with viral vectors require careful evaluation.

### 6.4 Cranial Bone Marrow Transport (CBM)

CBM is a novel technique derived from the tension‐stress theory proposed by the renowned medical expert Ilizarov in the 20th century. It involves the mobilization of cranial bone segments. In 2022, Lu et al. were the first to confirm that CBM can promote angiogenesis, enhance meningeal lymphatic drainage, and significantly accelerate recovery in rats with ischemic stroke [186]. In 2025, this team applied CBM to AD model mice, demonstrating that following the procedure, MLV generation was enhanced, meningeal lymphatic drainage improved, and memory function was restored [179].

However, significant limitations constrain the interpretability and translational potential of these findings. The CBM protocol itself was adapted from orthopedic applications and may not be optimal for neurological indications. Most critically, the precise mechanistic link between bone mobilization, meningeal lymphatic modulation, and functional recovery remains elusive, clouding its therapeutic rationale. Therefore, prior to any clinical consideration, fundamental studies must establish the optimal protocol, elucidate the underlying mechanisms, and evaluate long‐term efficacy and safety in translationally relevant models.

### 6.5 Lymphaticovenous Anastomosis (LVA)

Physical interventions targeting meningeal lymphatic drainage, particularly surgical techniques like LVA, represent a promising frontier in therapeutic exploration. This approach involves creating a microsurgical connection between lymphatic vessels and veins, most commonly in the deep cervical region, to enhance the physiological drainage of CSF and interstitial waste. Although still in its early stages, this method has been preliminarily applied in the treatment of AD. However, its clinical effectiveness and precise mechanism of action remain subjects of significant debate and scrutiny within the scientific community. To date, deep cervical LVA for AD has been predominantly investigated and performed in clinical research settings in China.

The rationale for this approach is partially supported by preclinical evidence. Animal studies have shown that surgically enhancing meningeal lymphatic function can alleviate cognitive deficits associated with pathological protein accumulation [189]. Building on this foundation, initial exploratory clinical studies have been conducted and provide preliminary human evidence consistent with the preclinical rationale. For instance, a prospective single‐arm study in 26 patients meeting NIA‐AA criteria for AD reported that deep cervical LVA was both safe and feasible. The study noted significant postoperative improvements in cognitive scores, such as the mini‐mental state examination (MMSE), though the authors stressed the need for long‐term follow‐up and larger controlled trials [203]. These early findings were further supported by a 2024 report on 50 patients with AD undergoing microsurgical LVA, which also documented significant improvements in MMSE and Montreal Cognitive Assessment (MoCA) scores [204].

Notably, the biological plausibility of this intervention has been bolstered by foundational research in nonhuman primates. Studies utilizing near‐infrared imaging have successfully visualized the dynamic drainage of CSF from the meningeal lymphatic system into dCLNs, providing critical in vivo evidence in a species physiologically closer to humans [205].

However, the relevance and interpretability of these early clinical data require cautious contextualization. To address the outstanding questions regarding efficacy and mechanism, rigorously designed clinical trials are now underway. Among these is the CLEAN‐AD trial (Identifier: NCT07073066), one of the first multicenter, randomized controlled studies on this topic, co‐designed by leading neurology and clinical trial methodology experts. The outcomes of these trials will be crucial to validate the preliminary cognitive benefits, elucidate the therapeutic mechanism, and determine the ultimate role of LVA in the therapeutic landscape for AD and related disorders.

## 7 Conclusion and Prospects

In conclusion, the identification of the glymphatic–lymphatic axis marks a foundational advancement in neuroscience, providing a mechanistic framework that links fundamental brain physiology to the pathogenesis of a broad spectrum of disorders. This review has demonstrated how this integrated system functions as the brain's primary waste clearance and immune surveillance conduit. Dysfunction of this axis—whether due to impaired glymphatic inflow, obstruction at critical junctures like the ACE points, or failure of meningeal lymphatic outflow—initiates and perpetuates a vicious cycle of protein accumulation, neuroinflammation, and neuronal damage. As observed in stroke, AD, and MS, although the specific mode of axis failure may vary, the result is consistently a disruption of CNS homeostasis that accelerates disease progression.

The therapeutic implications of this paradigm are profound and rapidly evolving. Strategies aimed at augmenting drainage dynamics, promoting lymphatic growth, modulating upstream drivers, or even surgically bypassing drainage obstacles are all aimed at restoring the integrity of this axis. However, as discussed, these interventions are double‐edged swords, with efficacy highly dependent on disease context, timing, and precision of delivery. The future of this field lies in moving beyond generic stimulation and toward precision modulation of specific axis components.

Several critical frontiers must be addressed to realize this potential. First, an urgent need exists to bridge the translational gap between rodent models and human biology. This includes validating imaging biomarkers, such as advanced MRI techniques, to dynamically assess axis function in patients and defining the human‐specific anatomy and regulatory biology of these structures. Second, future research must account for the temporal and spatial complexity of axis dysfunction. Therapeutic strategies must be tailored not only to the specific disease but also to its stage—whether it is an acute insult or chronic degeneration—and to the specific anatomical subsite that is compromised.

Finally, the greatest opportunity may lie in systems‐level integration. The glymphatic–lymphatic axis does not operate in isolation; it is modulated by sleep, systemic immunity, the systemic lymphatic system, and the cardiovascular system. The next generation of therapies may involve combination approaches that target multiple nodes or modulate lifestyle factors to synergistically restore clearance. Deepening our understanding of this intricate brain‐wide circuit will open a new frontier for developing effective interventions for some of the most challenging neurological diseases of our time.

## Author Contributions

We confirm that all authors have made substantial contributions to this work. In compliance with the latest guidelines of the International Committee of Medical Journal Editors (ICMJE), each author's specific contributions are as follows: Hangzhe Sun and Haonan Fan took charge of drawing the outline and drafting the manuscript. Yuhang Zhou and Haoliang Zhu drafted the figures. Yu Chen and Rui Zhang mainly made the tables. Anke Zhang, Kankai Wang, and Yuanbo Pan critically revised the manuscript for intellectual content and approved the final version. All authors have read and approved the final manuscript.

## Funding

This research was supported by the National Natural Science Foundation of China (82401598, 82571463) and Noncommunicable Chronic Diseases‐National Science and Technology Major Project (2023ZD0512800).

## Ethics Statement

The authors have nothing to report.

## Conflicts of Interest

The authors declare no conflicts of interest.

## Data Availability

Data and material sharing are not applicable to this article as no datasets or materials were generated or analyzed during the current study.

## References

[1] B. Engelhardt , P. Vajkoczy , and R. O. Weller , “The Movers and Shapers in Immune Privilege of the CNS,” Nature Immunology 18, no. 2 (2017): 123–131. 28092374 10.1038/ni.3666

[2] L. C. D. Smyth and J. Kipnis , “Redefining CNS Immune Privilege,” Nature Reviews Immunology 25, no. 10 (2025): 766–775. 10.1038/s41577-025-01175-0 40316862

[3] G. Ringstad , S. A. S. Vatnehol , and P. K. Eide , “Glymphatic MRI in Idiopathic Normal Pressure Hydrocephalus,” Brain: A Journal of Neurology 140, no. 10 (2017): 2691–2705. 28969373 10.1093/brain/awx191 PMC5841149

[4] J. J. Iliff , M. Wang , Y. Liao , et al., “A Paravascular Pathway Facilitates CSF Flow Through the Brain Parenchyma and the Clearance of Interstitial Solutes, Including Amyloid β,” Science Translational Medicine 4, no. 147 (2012): 147ra111. 10.1126/scitranslmed.3003748 PMC3551275 22896675

[5] A. Louveau , I. Smirnov , T. J. Keyes , et al., “Structural and Functional Features of Central Nervous System Lymphatic Vessels,” Nature 523, no. 7560 (2015): 337–341. 26030524 10.1038/nature14432 PMC4506234

[6] A. Aspelund , S. Antila , S. T. Proulx , et al., “A Dural Lymphatic Vascular System That Drains Brain Interstitial Fluid and Macromolecules,” Journal of Experimental Medicine 212, no. 7 (2015): 991–999. 26077718 10.1084/jem.20142290 PMC4493418

[7] M. K. Rasmussen , H. Mestre , and M. Nedergaard , “The Glymphatic Pathway in Neurological Disorders,” Lancet Neurology 17, no. 11 (2018): 1016–1024. 30353860 10.1016/S1474-4422(18)30318-1 PMC6261373

[8] A. F. M. Salvador , N. Abduljawad , and J. Kipnis , “Meningeal Lymphatics in Central Nervous System Diseases,” Annual Review of Neuroscience 47, no. 1 (2024): 323–344. 10.1146/annurev-neuro-113023-103045 PMC12051392 38648267

[9] M. Pollay , “The Function and Structure of the Cerebrospinal Fluid Outflow System,” Cerebrospinal Fluid Research 7 (2010): 9. 20565964 10.1186/1743-8454-7-9 PMC2904716

[10] R. Hodson , “Alzheimer's Disease,” Nature 559, no. 7715 (2018): S1. 30046078 10.1038/d41586-018-05717-6

[11] F. Gonzalez‐Ortiz , M. Turton , P. R. Kac , et al., “Brain‐Derived Tau: A Novel Blood‐Based Biomarker for Alzheimer's Disease‐Type Neurodegeneration,” Brain: A Journal of Neurology 146, no. 3 (2023): 1152–1165. 36572122 10.1093/brain/awac407 PMC9976981

[12] B. R. Bloem , M. S. Okun , and C. Klein , “Parkinson's Disease,” Lancet (London, England) 397, no. 10291 (2021): 2284–2303. 33848468 10.1016/S0140-6736(21)00218-X

[13] E. L. Feldman , S. A. Goutman , S. Petri , et al., “Amyotrophic Lateral Sclerosis,” Lancet (London, England) 400, no. 10360 (2022): 1363–1380. 36116464 10.1016/S0140-6736(22)01272-7 PMC10089700

[14] B. Song , Q. Ao , Z. Wang , et al., “Phosphorylation of Tau Protein Over Time in Rats Subjected to Transient Brain Ischemia,” Neural Regeneration Research 8, no. 34 (2013): 3173–3182. 25206638 10.3969/j.issn.1673-5374.2013.34.001 PMC4146185

[15] Y. Wen , S. Yang , R. Liu , and J. W. Simpkins , “Transient Cerebral Ischemia Induces Site‐Specific Hyperphosphorylation of Tau Protein,” Brain Research 1022, no. 1–2 (2004): 30–38. 15353210 10.1016/j.brainres.2004.05.106

[16] C. Jo , S. Gundemir , S. Pritchard , Y. N. Jin , I. Rahman , and G. V. Johnson , “Nrf2 Reduces Levels of Phosphorylated Tau Protein by Inducing Autophagy Adaptor Protein NDP52,” Nature Communications 5 (2014): 3496. 10.1038/ncomms4496 PMC3990284 24667209

[17] K. N. Corps , T. L. Roth , and D. B. McGavern , “Inflammation and Neuroprotection in Traumatic Brain Injury,” JAMA Neurology 72, no. 3 (2015): 355–362. 25599342 10.1001/jamaneurol.2014.3558 PMC5001842

[18] J. R. Casley‐Smith , E. Földi‐Börsök , and M. Földi , “The Prelymphatic Pathways of the Brain as Revealed by Cervical Lymphatic Obstruction and the Passage of Particles,” British Journal of Experimental Pathology 57, no. 2 (1976): 179–188. 773400 PMC2041107

[19] H. F. Cserr , D. N. Cooper , and T. H. Milhorat , “Flow of Cerebral Interstitial Fluid as Indicated by the Removal of Extracellular Markers From Rat Caudate Nucleus,” Experimental Eye Research 25, no. S1 (1977): 461–473. 590401 10.1016/s0014-4835(77)80041-9

[20] J. J. Iliff , H. Lee , M. Yu , et al., “Brain‐Wide Pathway for Waste Clearance Captured by Contrast‐Enhanced MRI,” Journal of Clinical Investigation 123, no. 3 (2013): 1299–1309. 23434588 10.1172/JCI67677 PMC3582150

[21] B. Bedussi , N. N. van der Wel , J. de Vos , et al., “Paravascular Channels, Cisterns, and the Subarachnoid Space in the Rat Brain: A Single Compartment With Preferential Pathways,” Journal of Cerebral Blood Flow and Metabolism: Official Journal of the International Society of Cerebral Blood Flow and Metabolism 37, no. 4 (2017): 1374–1385. 27306753 10.1177/0271678X16655550 PMC5453458

[22] B. A. Plog and M. Nedergaard , “The Glymphatic System in Central Nervous System Health and Disease: Past, Present, and Future,” Annual Review of Pathology 13 (2018): 379–394. 10.1146/annurev-pathol-051217-111018 PMC5803388 29195051

[23] M. L. Rennels , T. F. Gregory , O. R. Blaumanis , K. Fujimoto , and P. A. Grady , “Evidence for a ‘Paravascular’ Fluid Circulation in the Mammalian Central Nervous System, Provided by the Rapid Distribution of Tracer Protein Throughout the Brain From the Subarachnoid Space,” Brain Research 326, no. 1 (1985): 47–63. 3971148 10.1016/0006-8993(85)91383-6

[24] M. L. Rennels , O. R. Blaumanis , and P. A. Grady , “Rapid Solute Transport Throughout the Brain via Paravascular Fluid Pathways,” Advances in Neurology 52 (1990): 431–439. 2396537

[25] T. Ichimura , P. A. Fraser , and H. F. Cserr , “Distribution of Extracellular Tracers in Perivascular Spaces of the Rat Brain,” Brain Research 545, no. 1–2 (1991): 103–113. 1713524 10.1016/0006-8993(91)91275-6

[26] B. A. Plog , K. Kim , D. Verhaege , et al., “A Route for Cerebrospinal Fluid Flow Through Leptomeningeal Arterial‐Venous Overlaps Enables Macromolecule and Fluid Shunting,” Nature Neuroscience 28, no. 7 (2025): 1436–1445. 40490598 10.1038/s41593-025-01977-4

[27] S. Mader and L. Brimberg , “Aquaporin‐4 Water Channel in the Brain and Its Implication for Health and Disease,” Cells 8, no. 2 (2019): 90. 30691235 10.3390/cells8020090 PMC6406241

[28] M. Amiry‐Moghaddam , T. Otsuka , P. D. Hurn , et al., “An Alpha‐Syntrophin‐Dependent Pool of AQP4 in Astroglial End‐Feet Confers Bidirectional Water Flow Between Blood and Brain,” Proceedings of the National Academy of Sciences of the United States of America 100, no. 4 (2003): 2106–2111. 12578959 10.1073/pnas.0437946100 PMC149966

[29] L. Welberg , “Cognitive Neuroscience: Rules of Neural Engagement,” Nature Reviews Neuroscience 14, no. 1 (2013): 1. 10.1038/nrn3417 23232605

[30] E. A. Nagelhus and O. P. Ottersen , “Physiological Roles of Aquaporin‐4 in Brain,” Physiological Reviews 93, no. 4 (2013): 1543–1562. 24137016 10.1152/physrev.00011.2013 PMC3858210

[31] T. Nakada , I. L. Kwee , H. Igarashi , and Y. Suzuki , “Aquaporin‐4 Functionality and Virchow‐Robin Space Water Dynamics: Physiological Model for Neurovascular Coupling and Glymphatic Flow,” International Journal of Molecular Sciences 18, no. 8 (2017): 1798. 28820467 10.3390/ijms18081798 PMC5578185

[32] J. Cho , S. Lee , Y. H. Kook , et al., “Optogenetic Calcium Modulation in Astrocytes Enhances Post‐Stroke Recovery in Chronic Capsular Infarct,” Science Advances 11, no. 5 (2025): eadn7577. 39889003 10.1126/sciadv.adn7577 PMC11784845

[33] M. J. Giannetto , R. S. Gomolka , D. Gahn‐Martinez , et al., “Glymphatic Fluid Transport Is Suppressed by the Aquaporin‐4 Inhibitor AER‐271,” Glia 72, no. 5 (2024): 982–998. 38363040 10.1002/glia.24515 PMC11203403

[34] I. Lundgaard , B. Li , L. Xie , et al., “Direct Neuronal Glucose Uptake Heralds Activity‐Dependent Increases in Cerebral Metabolism,” Nature Communications 6 (2015): 6807. 10.1038/ncomms7807 PMC4410436 25904018

[35] V. Rangroo Thrane , A. S. Thrane , B. A. Plog , et al., “Paravascular Microcirculation Facilitates Rapid Lipid Transport and Astrocyte Signaling in the Brain,” Scientific Reports 3 (2013): 2582. 24002448 10.1038/srep02582 PMC3761080

[36] L. Xie , H. Kang , Q. Xu , et al., “Sleep Drives Metabolite Clearance From the Adult Brain,” Science (New York, NY) 342, no. 6156 (2013): 373–377. 10.1126/science.1241224 PMC3880190 24136970

[37] J. J. Iliff , M. Wang , D. M. Zeppenfeld , et al., “Cerebral Arterial Pulsation Drives Paravascular CSF‐Interstitial Fluid Exchange in the Murine Brain,” Journal of Neuroscience: The Official Journal of the Society for Neuroscience 33, no. 46 (2013): 18190–18199. 24227727 10.1523/JNEUROSCI.1592-13.2013 PMC3866416

[38] K. Hochrainer , K. Jackman , C. Benakis , J. Anrather , and C. Iadecola , “SUMO2/3 is Associated With Ubiquitinated Protein Aggregates in the Mouse Neocortex After Middle Cerebral Artery Occlusion,” Journal of Cerebral Blood Flow and Metabolism: Official Journal of the International Society of Cerebral Blood Flow and Metabolism 35, no. 1 (2015): 1–5. 25352045 10.1038/jcbfm.2014.180 PMC4294403

[39] R. T. Kedarasetti , P. J. Drew , and F. Costanzo , “Arterial Pulsations Drive Oscillatory Flow of CSF but Not Directional Pumping,” Scientific Reports 10, no. 1 (2020): 10102. 32572120 10.1038/s41598-020-66887-w PMC7308311

[40] H. Lee , L. Xie , M. Yu , et al., “The Effect of Body Posture on Brain Glymphatic Transport,” Journal of Neuroscience: The Official Journal of the Society for Neuroscience 35, no. 31 (2015): 11034–11044. 26245965 10.1523/JNEUROSCI.1625-15.2015 PMC4524974

[41] L. Ladriere , T. M. Zhang , and W. J. Malaisse , “Effects of Succinic Acid Dimethyl Ester Infusion on Metabolic, Hormonal, and Enzymatic Variables in Starved Rats,” JPEN Journal of Parenteral and Enteral Nutrition 20, no. 4 (1996): 251–256. 8865105 10.1177/0148607196020004251

[42] J. B. Murphy and E. Sturm , “Conditions Determining the Transplantability of Tissues in the Brain,” Journal of Experimental Medicine 38, no. 2 (1923): 183–197. 19868782 10.1084/jem.38.2.183 PMC2128434

[43] P. B. Medawar , “Immunological Tolerance,” Nature 189 (1961): 14–17. 13768821 10.1038/189014a0

[44] S. Sandrone , D. Moreno‐Zambrano , J. Kipnis , and J. van Gijn , “A (Delayed) History of the Brain Lymphatic System,” Nature Medicine 25, no. 4 (2019): 538–540. 10.1038/s41591-019-0417-3 30948855

[45] J. Li , J. Zhou , and Y. Shi , “Scanning Electron Microscopy of Human Cerebral Meningeal Stomata,” Annals of Anatomy = Anatomischer Anzeiger: Official Organ of the Anatomische Gesellschaft 178, no. 3 (1996): 259–261. 8712374 10.1016/S0940-9602(96)80059-8

[46] N. C. Derecki , A. N. Cardani , C. H. Yang , et al., “Regulation of Learning and Memory by Meningeal Immunity: A Key Role for IL‐4,” Journal of Experimental Medicine 207, no. 5 (2010): 1067–1080. 20439540 10.1084/jem.20091419 PMC2867291

[47] C. L. Vera Quesada , S. B. Rao , R. Torp , and P. K. Eide , “Widespread Distribution of Lymphatic Vessels in Human Dura Mater Remote From Sinus Veins,” Frontiers in Cell and Developmental Biology 11 (2023): 1228344. 37795263 10.3389/fcell.2023.1228344 PMC10546208

[48] P. Baluk , J. Fuxe , H. Hashizume , et al., “Functionally Specialized Junctions Between Endothelial Cells of Lymphatic Vessels,” Journal of Experimental Medicine 204, no. 10 (2007): 2349–2362. 17846148 10.1084/jem.20062596 PMC2118470

[49] T. V. Petrova and G. Y. Koh , “Organ‐Specific Lymphatic Vasculature: From Development to Pathophysiology,” Journal of Experimental Medicine 215, no. 1 (2018): 35–49. 29242199 10.1084/jem.20171868 PMC5748863

[50] K. N. Margaris and R. A. Black , “Modelling the Lymphatic System: Challenges and Opportunities,” Journal of the Royal Society, Interface 9, no. 69 (2012): 601–612. 22237677 10.1098/rsif.2011.0751 PMC3284143

[51] J. H. Ahn , H. Cho , J. H. Kim , et al., “Meningeal Lymphatic Vessels at the Skull Base Drain Cerebrospinal Fluid,” Nature 572, no. 7767 (2019): 62–66. 31341278 10.1038/s41586-019-1419-5

[52] J. H. Yoon , H. Jin , H. J. Kim , et al., “Nasopharyngeal Lymphatic Plexus Is a Hub for Cerebrospinal Fluid Drainage,” Nature 625, no. 7996 (2024): 768–777. 38200313 10.1038/s41586-023-06899-4 PMC10808075

[53] Y. Decker , J. Krämer , L. Xin , et al., “Magnetic Resonance Imaging of Cerebrospinal Fluid Outflow After Low‐Rate Lateral Ventricle Infusion in Mice,” JCI Insight 7, no. 3 (2022): e150881. 34905509 10.1172/jci.insight.150881 PMC8855808

[54] J. N. Norwood , Q. Zhang , D. Card , A. Craine , T. M. Ryan , and P. J. Drew , “Anatomical Basis and Physiological Role of Cerebrospinal Fluid Transport Through the Murine Cribriform Plate,” eLife 8 (2019): e44278. 31063132 10.7554/eLife.44278 PMC6524970

[55] L. Jacob , J. de Brito Neto , S. Lenck , et al., “Conserved Meningeal Lymphatic Drainage Circuits in Mice and Humans,” Journal of Experimental Medicine 219, no. 8 (2022): e20220035. 35776089 10.1084/jem.20220035 PMC9253621

[56] S. Shibata‐Germanos , J. R. Goodman , A. Grieg , et al., “Structural and Functional Conservation of Non‐Lumenized Lymphatic Endothelial Cells in the Mammalian Leptomeninges,” Acta Neuropathologica 139, no. 2 (2020): 383–401. 31696318 10.1007/s00401-019-02091-z PMC6989586

[57] R. Cai , C. Pan , A. Ghasemigharagoz , et al., “Panoptic Imaging of Transparent Mice Reveals Whole‐Body Neuronal Projections and Skull‐Meninges Connections,” Nature Neuroscience 22, no. 2 (2019): 317–327. 30598527 10.1038/s41593-018-0301-3 PMC6494982

[58] A. Tamadon , A. Afshar , N. M. Mussin , et al., “Mouse Brain Lymphatic Vessels,” ACS Chemical Neuroscience 16, no. 23 (2025): 4492–4501. 41263424 10.1021/acschemneuro.5c00533

[59] Q. Zhang , Y. Niu , Y. Li , et al., “Meningeal Lymphatic Drainage: Novel Insights Into Central Nervous System Disease,” Signal Transduction and Targeted Therapy 10, no. 1 (2025): 142. 40320416 10.1038/s41392-025-02177-z PMC12050339

[60] N. Delivanoglou and S. Da Mesquita , “CNS‐Draining Meningeal Lymphatic Vasculature: Roles, Conundrums and Future Challenges,” Frontiers in Pharmacology 12 (2021): 655052. 33995074 10.3389/fphar.2021.655052 PMC8113819

[61] M. L. Upton and R. O. Weller , “The Morphology of Cerebrospinal Fluid Drainage Pathways in Human Arachnoid Granulations,” Journal of Neurosurgery 63, no. 6 (1985): 867–875. 4056901 10.3171/jns.1985.63.6.0867

[62] S. Kida , A. Pantazis , and R. O. Weller , “CSF Drains Directly From the Subarachnoid Space Into Nasal Lymphatics in the Rat. Anatomy, Histology and Immunological Significance,” Neuropathology and Applied Neurobiology 19, no. 6 (1993): 480–488. 7510047 10.1111/j.1365-2990.1993.tb00476.x

[63] M. W. Bradbury and R. J. Westrop , “Factors Influencing Exit of Substances From Cerebrospinal Fluid Into Deep Cervical Lymph of the Rabbit,” Journal of Physiology 339 (1983): 519–534. 6411905 10.1113/jphysiol.1983.sp014731 PMC1199176

[64] S. Antila , S. Karaman , H. Nurmi , et al., “Development and Plasticity of Meningeal Lymphatic Vessels,” Journal of Experimental Medicine 214, no. 12 (2017): 3645–3667. 29141865 10.1084/jem.20170391 PMC5716035

[65] M. W. Bradbury and D. F. Cole , “The Role of the Lymphatic System in Drainage of Cerebrospinal Fluid and Aqueous Humour,” Journal of Physiology 299 (1980): 353–365. 6155466 10.1113/jphysiol.1980.sp013129 PMC1279229

[66] L. C. D. Smyth , D. Xu , S. V. Okar , et al., “Identification of Direct Connections Between the Dura and the Brain,” Nature 627, no. 8002 (2024): 165–173. 38326613 10.1038/s41586-023-06993-7 PMC11254388

[67] Q. Ma , Y. Decker , A. Müller , B. V. Ineichen , and S. T. Proulx , “Clearance of Cerebrospinal Fluid From the Sacral Spine Through Lymphatic Vessels,” Journal of Experimental Medicine 216, no. 11 (2019): 2492–2502. 31455602 10.1084/jem.20190351 PMC6829589

[68] L. Jacob , L. S. B. Boisserand , L. H. M. Geraldo , et al., “Anatomy and Function of the Vertebral Column Lymphatic Network in Mice,” Nature Communications 10, no. 1 (2019): 4594. 10.1038/s41467-019-12568-w PMC6785564 31597914

[69] H. F. Cserr , C. J. Harling‐Berg , and P. M. Knopf , “Drainage of Brain Extracellular Fluid Into Blood and Deep Cervical Lymph and Its Immunological Significance,” Brain Pathology (Zurich, Switzerland) 2, no. 4 (1992): 269–276. 1341962 10.1111/j.1750-3639.1992.tb00703.x

[70] X. Liang and H. Luo , “Optical Tissue Clearing: Illuminating Brain Function and Dysfunction,” Theranostics 11, no. 7 (2021): 3035–3051. 33537072 10.7150/thno.53979 PMC7847687

[71] D. M. McDonald , K. Alitalo , C. Betsholtz , et al., “Cerebrospinal Fluid Draining Lymphatics in Health and Disease: Advances and Controversies,” Nature Cardiovascular Research 4, no. 9 (2025): 1047–1065. 10.1038/s44161-025-00705-2 40921861

[72] S. Muralidar , S. V. Ambi , S. Sekaran , D. Thirumalai , and B. Palaniappan , “Role of Tau Protein in Alzheimer's Disease: The Prime Pathological Player,” International Journal of Biological Macromolecules 163 (2020): 1599–1617. 32784025 10.1016/j.ijbiomac.2020.07.327

[73] H. W. Querfurth and F. M. LaFerla , “Alzheimer's Disease,” New England Journal of Medicine 362, no. 4 (2010): 329–344. 20107219 10.1056/NEJMra0909142

[74] D. M. Lopes , S. K. Llewellyn , and I. F. Harrison , “Propagation of Tau and α‐Synuclein in the Brain: Therapeutic Potential of the Glymphatic System,” Translational Neurodegeneration 11, no. 1 (2022): 19. 35314000 10.1186/s40035-022-00293-2 PMC8935752

[75] Y. R. Wen , J. H. Yang , X. Wang , and Z. B. Yao , “Induced Dural Lymphangiogenesis Facilities Soluble Amyloid‐Beta Clearance From Brain in a Transgenic Mouse Model of Alzheimer's Disease,” Neural Regeneration Research 13, no. 4 (2018): 709–716. 29722325 10.4103/1673-5374.230299 PMC5950683

[76] S. Da Mesquita , A. Louveau , and A. Vaccari , “Functional Aspects of Meningeal Lymphatics in Ageing and Alzheimer's Disease,” Nature 560, no. 7717 (2018): 185–191. 30046111 10.1038/s41586-018-0368-8 PMC6085146

[77] T. K. Patel , L. Habimana‐Griffin , X. Gao , et al., “Dural Lymphatics Regulate Clearance of Extracellular Tau From the CNS,” Molecular Neurodegeneration 14, no. 1 (2019): 11. 30813965 10.1186/s13024-019-0312-x PMC6391770

[78] X. B. Ding , X. X. Wang , D. H. Xia , et al., “Impaired Meningeal Lymphatic Drainage in Patients With Idiopathic Parkinson's Disease,” Nature Medicine 27, no. 3 (2021): 411–418. 10.1038/s41591-020-01198-1 33462448

[79] M. Liu , J. Huang , T. Liu , et al., “Exogenous Interleukin 33 Enhances the Brain's Lymphatic Drainage and Toxic Protein Clearance in Acute Traumatic Brain Injury Mice,” Acta Neuropathologica Communications 11, no. 1 (2023): 61. 37024941 10.1186/s40478-023-01555-4 PMC10080777

[80] J. Liao , M. Zhang , Z. Shi , et al., “Improving the Function of Meningeal Lymphatic Vessels to Promote Brain Edema Absorption After Traumatic Brain Injury,” Journal of Neurotrauma 40, no. 3–4 (2023): 383–394. 36106596 10.1089/neu.2022.0150

[81] J. Chen , L. Wang , H. Xu , et al., “Meningeal Lymphatics Clear Erythrocytes That Arise From Subarachnoid Hemorrhage,” Nature Communications 11, no. 1 (2020): 3159. 10.1038/s41467-020-16851-z PMC7308412 32572022

[82] R. Rua and D. B. McGavern , “Advances in Meningeal Immunity,” Trends in Molecular Medicine 24, no. 6 (2018): 542–559. 29731353 10.1016/j.molmed.2018.04.003 PMC6044730

[83] J. Kipnis , “Multifaceted Interactions Between Adaptive Immunity and the Central Nervous System,” Science (New York, NY) 353, no. 6301 (2016): 766–771. 10.1126/science.aag2638 PMC5590839 27540163

[84] G. T. Norris and J. Kipnis , “Immune Cells and CNS Physiology: Microglia and Beyond,” Journal of Experimental Medicine 216, no. 1 (2019): 60–70. 30504438 10.1084/jem.20180199 PMC6314530

[85] D. Mrdjen , A. Pavlovic , F. J. Hartmann , et al., “High‐Dimensional Single‐Cell Mapping of Central Nervous System Immune Cells Reveals Distinct Myeloid Subsets in Health, Aging, and Disease,” Immunity 48, no. 2 (2018): 380–395.e6. 29426702 10.1016/j.immuni.2018.01.011

[86] J. Kipnis , H. Benveniste , A. Eichmann , et al., “Resolving the Mysteries of Brain Clearance and Immune Surveillance,” Neuron 113, no. 23 (2025): 3908–3923. 41289996 10.1016/j.neuron.2025.10.036 PMC12757055

[87] A. Louveau , J. Herz , M. N. Alme , et al., “CNS Lymphatic Drainage and Neuroinflammation Are Regulated by Meningeal Lymphatic Vasculature,” Nature Neuroscience 21, no. 10 (2018): 1380–1391. 30224810 10.1038/s41593-018-0227-9 PMC6214619

[88] H. Van Hove , L. Martens , I. Scheyltjens , et al., “A Single‐Cell Atlas of Mouse Brain Macrophages Reveals Unique Transcriptional Identities Shaped by Ontogeny and Tissue Environment,” Nature Neuroscience 22, no. 6 (2019): 1021–1035. 31061494 10.1038/s41593-019-0393-4

[89] R. Daneman , L. Zhou , A. A. Kebede , and B. A. Barres , “Pericytes Are Required for Blood‐Brain Barrier Integrity During Embryogenesis,” Nature 468, no. 7323 (2010): 562–566. 20944625 10.1038/nature09513 PMC3241506

[90] S. Brioschi , W. L. Wang , V. Peng , et al., “Heterogeneity of Meningeal B Cells Reveals a Lymphopoietic Niche at the CNS Borders,” Science (New York, NY) 373, no. 6553 (2021): eabf9277. 10.1126/science.abf9277 PMC8448524 34083450

[91] J. Rustenhoven , A. Drieu , T. Mamuladze , et al., “Functional Characterization of the Dural Sinuses as a Neuroimmune Interface,” Cell 184, no. 4 (2021): 1000–1016.e27. 33508229 10.1016/j.cell.2020.12.040 PMC8487654

[92] Q. Zhang , Y. Chen , Y. Li , et al., “Neutrophil Extracellular Trap‐Mediated Impairment of Meningeal Lymphatic Drainage Exacerbates Secondary Hydrocephalus After Intraventricular Hemorrhage,” Theranostics 14, no. 5 (2024): 1909–1938. 38505607 10.7150/thno.91653 PMC10945341

[93] A. Drieu , S. Du , S. E. Storck , et al., “Parenchymal Border Macrophages Regulate the Flow Dynamics of the Cerebrospinal Fluid,” Nature 611, no. 7936 (2022): 585–593. 36352225 10.1038/s41586-022-05397-3 PMC9899827

[94] M. Absinta , S. K. Ha , G. Nair , et al., “Human and Nonhuman Primate Meninges Harbor Lymphatic Vessels That Can be Visualized Noninvasively by MRI,” eLife 6 (2017): e29738. 28971799 10.7554/eLife.29738 PMC5626482

[95] D. Castranova , B. Samasa , M. Venero Galanternik , H. M. Jung , V. N. Pham , and B. M. Weinstein , “Live Imaging of Intracranial Lymphatics in the Zebrafish,” Circulation Research 128, no. 1 (2021): 42–58. 33135960 10.1161/CIRCRESAHA.120.317372 PMC7790877

[96] J. R. Goodman , Z. O. Adham , R. L. Woltjer , A. W. Lund , and J. J. Iliff , “Characterization of Dural Sinus‐Associated Lymphatic Vasculature in Human Alzheimer's Dementia Subjects,” Brain, Behavior, and Immunity 73 (2018): 34–40. 30055243 10.1016/j.bbi.2018.07.020 PMC6149215

[97] M. Park , J. W. Kim , S. J. Ahn , Y. J. Cha , and S. H. Suh , “Aging Is Positively Associated With Peri‐Sinus Lymphatic Space Volume: Assessment Using 3T Black‐Blood MRI,” Journal of Clinical Medicine 9, no. 10 (2020): 3353. 33086702 10.3390/jcm9103353 PMC7590154

[98] M. van Lessen , S. Shibata‐Germanos , A. van Impel , T. A. Hawkins , J. Rihel , and S. Schulte‐Merker , “Intracellular Uptake of Macromolecules by Brain Lymphatic Endothelial Cells During Zebrafish Embryonic Development,” eLife 6 (2017): e25932. 28498105 10.7554/eLife.25932 PMC5457137

[99] E. Candelario‐Jalil , R. M. Dijkhuizen , and T. Magnus , “Neuroinflammation, Stroke, Blood‐Brain Barrier Dysfunction, and Imaging Modalities,” Stroke 53, no. 5 (2022): 1473–1486. 35387495 10.1161/STROKEAHA.122.036946 PMC9038693

[100] D. Singh , “Astrocytic and Microglial Cells as the Modulators of Neuroinflammation in Alzheimer's Disease,” Journal of Neuroinflammation 19, no. 1 (2022): 206. 35978311 10.1186/s12974-022-02565-0 PMC9382837

[101] W. Zhang , D. Xiao , Q. Mao , and H. Xia , “Role of Neuroinflammation in Neurodegeneration Development,” Signal Transduction and Targeted Therapy 8, no. 1 (2023): 267. 37433768 10.1038/s41392-023-01486-5 PMC10336149

[102] R. L. Jayaraj , S. Azimullah , R. Beiram , F. Y. Jalal , and G. A. Rosenberg , “Neuroinflammation: Friend and Foe for Ischemic Stroke,” Journal of Neuroinflammation 16, no. 1 (2019): 142. 31291966 10.1186/s12974-019-1516-2 PMC6617684

[103] D. L. Alsbrook , M. Di Napoli , K. Bhatia , et al., “Neuroinflammation in Acute Ischemic and Hemorrhagic Stroke,” Current Neurology and Neuroscience Reports 23, no. 8 (2023): 407–431. 37395873 10.1007/s11910-023-01282-2 PMC10544736

[104] Y. Ma , Y. Liu , Z. Zhang , and G. Y. Yang , “Significance of Complement System in Ischemic Stroke: A Comprehensive Review,” Aging and Disease 10, no. 2 (2019): 429–462. 31011487 10.14336/AD.2019.0119 PMC6457046

[105] E. D. Pedersen , U. Waje‐Andreassen , C. A. Vedeler , G. Aamodt , and T. E. Mollnes , “Systemic Complement Activation Following Human Acute Ischaemic Stroke,” Clinical and Experimental Immunology 137, no. 1 (2004): 117–122. 15196251 10.1111/j.1365-2249.2004.02489.x PMC1809093

[106] A. Lampron , A. Elali , and S. Rivest , “Innate Immunity in the CNS: Redefining the Relationship Between the CNS and Its Environment,” Neuron 78, no. 2 (2013): 214–232. 23622060 10.1016/j.neuron.2013.04.005

[107] A. Waisman , R. S. Liblau , and B. Becher , “Innate and Adaptive Immune Responses in the CNS,” Lancet Neurology 14, no. 9 (2015): 945–955. 26293566 10.1016/S1474-4422(15)00141-6

[108] L. Y. Zhang , J. Pan , M. Mamtilahun , et al., “Microglia Exacerbate White Matter Injury via Complement C3/C3aR Pathway After Hypoperfusion,” Theranostics 10, no. 1 (2020): 74–90. 31903107 10.7150/thno.35841 PMC6929610

[109] A. Jurcau and A. Simion , “Neuroinflammation in Cerebral Ischemia and Ischemia/Reperfusion Injuries: From Pathophysiology to Therapeutic Strategies,” International Journal of Molecular Sciences 23, no. 1 (2021): 14. 35008440 10.3390/ijms23010014 PMC8744548

[110] X. Shui , J. Chen , Z. Fu , H. Zhu , H. Tao , and Z. Li , “Microglia in Ischemic Stroke: Pathogenesis Insights and Therapeutic Challenges,” Journal of Inflammation Research 17 (2024): 3335–3352. 38800598 10.2147/JIR.S461795 PMC11128258

[111] A. Sierra , O. Abiega , A. Shahraz , and H. Neumann , “Janus‐Faced Microglia: Beneficial and Detrimental Consequences of Microglial Phagocytosis,” Frontiers in Cellular Neuroscience 7 (2013): 6. 23386811 10.3389/fncel.2013.00006 PMC3558702

[112] S. Xu , J. Lu , A. Shao , J. H. Zhang , and J. Zhang , “Glial Cells: Role of the Immune Response in Ischemic Stroke,” Frontiers in Immunology 11 (2020): 294. 32174916 10.3389/fimmu.2020.00294 PMC7055422

[113] C. Qin , S. Yang , Y. H. Chu , et al., “Signaling Pathways Involved in Ischemic Stroke: Molecular Mechanisms and Therapeutic Interventions,” Signal Transduction and Targeted Therapy 7, no. 1 (2022): 215. 35794095 10.1038/s41392-022-01064-1 PMC9259607

[114] R. Dong , R. Huang , J. Wang , H. Liu , and Z. Xu , “Effects of Microglial Activation and Polarization on Brain Injury After Stroke,” Frontiers in Neurology 12 (2021): 620948. 34276530 10.3389/fneur.2021.620948 PMC8280287

[115] R. C. Paolicelli , A. Sierra , B. Stevens , et al., “Microglia States and Nomenclature: A Field at Its Crossroads,” Neuron 110, no. 21 (2022): 3458–3483. 36327895 10.1016/j.neuron.2022.10.020 PMC9999291

[116] M. Wang , Y. Hua , R. F. Keep , S. Wan , N. Novakovic , and G. Xi , “Complement Inhibition Attenuates Early Erythrolysis in the Hematoma and Brain Injury in Aged Rats,” Stroke 50, no. 7 (2019): 1859–1868. 31177985 10.1161/STROKEAHA.119.025170 PMC6591097

[117] R. Jin , L. Liu , S. Zhang , A. Nanda , and G. Li , “Role of Inflammation and Its Mediators in Acute Ischemic Stroke,” Journal of Cardiovascular Translational Research 6, no. 5 (2013): 834–851. 24006091 10.1007/s12265-013-9508-6 PMC3829610

[118] M. M. Varnum , T. Kiyota , K. L. Ingraham , S. Ikezu , and T. Ikezu , “The Anti‐Inflammatory Glycoprotein, CD200, Restores Neurogenesis and Enhances Amyloid Phagocytosis in a Mouse Model of Alzheimer's Disease,” Neurobiology of Aging 36, no. 11 (2015): 2995–3007. 26315370 10.1016/j.neurobiolaging.2015.07.027 PMC4772879

[119] X. Y. Shen , Z. K. Gao , Y. Han , M. Yuan , Y. S. Guo , and X. Bi , “Activation and Role of Astrocytes in Ischemic Stroke,” Frontiers in Cellular Neuroscience 15 (2021): 755955. 34867201 10.3389/fncel.2021.755955 PMC8635513

[120] C. Liu , Y. Guo , S. Deng , et al., “Hemorrhagic Stroke‐Induced Subtype of Inflammatory Reactive Astrocytes Disrupts Blood‐Brain Barrier,” Journal of Cerebral Blood Flow and Metabolism: Official Journal of the International Society of Cerebral Blood Flow and Metabolism 44, no. 7 (2024): 1102–1116. 38388375 10.1177/0271678X241235008 PMC11179611

[121] N. Blank‐Stein and E. Mass , “Macrophage and Monocyte Subsets in Response to Ischemic Stroke,” European Journal of Immunology 53, no. 10 (2023): e2250233. 37467166 10.1002/eji.202250233

[122] J. Pedragosa , A. Salas‐Perdomo , M. Gallizioli , et al., “CNS‐Border Associated Macrophages Respond to Acute Ischemic Stroke Attracting Granulocytes and Promoting Vascular Leakage,” Acta Neuropathologica Communications 6, no. 1 (2018): 76. 30092836 10.1186/s40478-018-0581-6 PMC6083589

[123] K. Zheng , L. Lin , W. Jiang , et al., “Single‐Cell RNA‐Seq Reveals the Transcriptional Landscape in Ischemic Stroke,” Journal of Cerebral Blood Flow and Metabolism: Official Journal of the International Society of Cerebral Blood Flow and Metabolism 42, no. 1 (2022): 56–73. 34496660 10.1177/0271678X211026770 PMC8721774

[124] E. Kim and S. Cho , “Microglia and Monocyte‐Derived Macrophages in Stroke,” Neurotherapeutics: The Journal of the American Society for Experimental NeuroTherapeutics 13, no. 4 (2016): 702–718. 27485238 10.1007/s13311-016-0463-1 PMC5081116

[125] R. M. Ransohoff and A. E. Cardona , “The Myeloid Cells of the Central Nervous System Parenchyma,” Nature 468, no. 7321 (2010): 253–262. 21068834 10.1038/nature09615

[126] C. Meisel , J. M. Schwab , K. Prass , A. Meisel , and U. Dirnagl , “Central Nervous System Injury‐Induced Immune Deficiency Syndrome,” Nature Reviews Neuroscience 6, no. 10 (2005): 775–786. 16163382 10.1038/nrn1765

[127] M. Endres , M. A. Moro , C. H. Nolte , C. Dames , M. S. Buckwalter , and A. Meisel , “Immune Pathways in Etiology, Acute Phase, and Chronic Sequelae of Ischemic Stroke,” Circulation Research 130, no. 8 (2022): 1167–1186. 35420915 10.1161/CIRCRESAHA.121.319994

[128] P. Carmona‐Mora , B. Knepp , G. C. Jickling , et al., “Monocyte, Neutrophil, and Whole Blood Transcriptome Dynamics Following Ischemic Stroke,” BMC Medicine 21, no. 1 (2023): 65. 36803375 10.1186/s12916-023-02766-1 PMC9942321

[129] I. Bartholomäus , N. Kawakami , F. Odoardi , et al., “Effector T Cell Interactions With Meningeal Vascular Structures in Nascent Autoimmune CNS Lesions,” Nature 462, no. 7269 (2009): 94–98. 19829296 10.1038/nature08478

[130] Y. Feng , S. Liao , C. Wei , et al., “Infiltration and Persistence of Lymphocytes During Late‐Stage Cerebral Ischemia in Middle Cerebral Artery Occlusion and Photothrombotic Stroke Models,” Journal of Neuroinflammation 14, no. 1 (2017): 248. 29246244 10.1186/s12974-017-1017-0 PMC5732427

[131] A. Liesz , X. Hu , C. Kleinschnitz , and H. Offner , “Functional Role of Regulatory Lymphocytes in Stroke: Facts and Controversies,” Stroke 46, no. 5 (2015): 1422–1430, 10.1161/strokeaha.114.008608. 25791715 PMC4414876

[132] Z. Lužnik , S. Anchouche , R. Dana , and J. Yin , “Regulatory T Cells in Angiogenesis,” Journal of Immunology (Baltimore, Md: 1950) 205, no. 10 (2020): 2557–2565. 33168598 10.4049/jimmunol.2000574 PMC7664842

[133] S. B. Ortega , V. O. Torres , S. E. Latchney , et al., “B Cells Migrate Into Remote Brain Areas and Support Neurogenesis and Functional Recovery After Focal Stroke in Mice,” Proceedings of the National Academy of Sciences of the United States of America 117, no. 9 (2020): 4983–4993. 32051245 10.1073/pnas.1913292117 PMC7060723

[134] X. Wang , A. Zhang , Q. Yu , et al., “Single‐Cell RNA Sequencing and Spatial Transcriptomics Reveal Pathogenesis of Meningeal Lymphatic Dysfunction After Experimental Subarachnoid Hemorrhage,” Advanced Science (Weinheim, Baden‐Wurttemberg, Germany) 10, no. 21 (2023): e2301428. 37211686 10.1002/advs.202301428 PMC10375135

[135] H. H. Tsai , Y. C. Hsieh , J. S. Lin , et al., “Functional Investigation of Meningeal Lymphatic System in Experimental Intracerebral Hemorrhage,” Stroke 53, no. 3 (2022): 987–998. 35144488 10.1161/STROKEAHA.121.037834

[136] H. Pawluk , A. Woźniak , G. Grześk , et al., “The Role of Selected Pro‐Inflammatory Cytokines in Pathogenesis of Ischemic Stroke,” Clinical Interventions in Aging 15 (2020): 469–484. 32273689 10.2147/CIA.S233909 PMC7110925

[137] Y. Cai , Y. Shao , H. Yuan , et al., “Meningeal Lymphatic Dysfunction Drives Cognitive Impairment After Experimental Subarachnoid Hemorrhage,” Neurotherapeutics: The Journal of the American Society for Experimental NeuroTherapeutics 23, no. 1 (2025): e00819. 41390288 10.1016/j.neurot.2025.e00819 PMC12976478

[138] M. Hsu , C. Laaker , A. Madrid , et al., “Neuroinflammation Creates an Immune Regulatory Niche at the Meningeal Lymphatic Vasculature Near the Cribriform Plate,” Nature Immunology 23, no. 4 (2022): 581–593. 35347285 10.1038/s41590-022-01158-6 PMC8989656

[139] R. Shimada , Y. Tatara , and K. Kibayashi , “Gene Expression in Meningeal Lymphatic Endothelial Cells Following Traumatic Brain Injury in Mice,” PloS ONE 17, no. 9 (2022): e0273892. 36067135 10.1371/journal.pone.0273892 PMC9447870

[140] P. Miossec and J. K. Kolls , “Targeting IL‐17 and TH17 Cells in Chronic Inflammation,” Nature Reviews Drug Discovery 11, no. 10 (2012): 763–776. 23023676 10.1038/nrd3794

[141] S. P. das Neves , N. Delivanoglou , Y. Ren , et al., “Meningeal Lymphatic Function Promotes Oligodendrocyte Survival and Brain Myelination,” Immunity 57, no. 10 (2024): 2328–2343.e8. 39217987 10.1016/j.immuni.2024.08.004 PMC11464205

[142] E. Esposito , B. J. Ahn , J. Shi , et al., “Brain‐To‐Cervical Lymph Node Signaling After Stroke,” Nature Communications 10, no. 1 (2019): 5306. 10.1038/s41467-019-13324-w PMC6876639 31757960

[143] H. Kim , R. P. Kataru , and G. Y. Koh , “Inflammation‐Associated Lymphangiogenesis: A Double‐Edged Sword?,” Journal of Clinical Investigation 124, no. 3 (2014): 936–942. 24590279 10.1172/JCI71607 PMC3938274

[144] S. Chen , L. Shao , and L. Ma , “Cerebral Edema Formation After Stroke: Emphasis on Blood‐Brain Barrier and the Lymphatic Drainage System of the Brain,” Frontiers in Cellular Neuroscience 15 (2021): 716825. 34483842 10.3389/fncel.2021.716825 PMC8415457

[145] K. T. Kahle , J. M. Simard , K. J. Staley , B. V. Nahed , P. S. Jones , and D. Sun , “Molecular Mechanisms of Ischemic Cerebral Edema: Role of Electroneutral Ion Transport,” Physiology (Bethesda, Md) 24 (2009): 257–265. 19675357 10.1152/physiol.00015.2009

[146] K. G. Holste , F. Xia , F. Ye , R. F. Keep , and G. Xi , “Mechanisms of Neuroinflammation in Hydrocephalus After Intraventricular Hemorrhage: A Review,” Fluids and Barriers of the CNS 19, no. 1 (2022): 28. 35365172 10.1186/s12987-022-00324-0 PMC8973639

[147] H. Mestre , T. Du , A. M. Sweeney , et al., “Cerebrospinal Fluid Influx Drives Acute Ischemic Tissue Swelling,” Science (New York, NY) 367, no. 6483 (2020): eaax7171. 10.1126/science.aax7171 PMC7375109 32001524

[148] B. I. Koh , H. J. Lee , P. A. Kwak , et al., “VEGFR2 Signaling Drives Meningeal Vascular Regeneration Upon Head Injury,” Nature Communications 11, no. 1 (2020): 3866. 10.1038/s41467-020-17545-2 PMC7395111 32737287

[149] P. Yanev , K. Poinsatte , D. Hominick , et al., “Impaired Meningeal Lymphatic Vessel Development Worsens Stroke Outcome,” Journal of Cerebral Blood Flow and Metabolism: Official Journal of the International Society of Cerebral Blood Flow and Metabolism 40, no. 2 (2020): 263–275. 30621519 10.1177/0271678X18822921 PMC7370617

[150] J. Chen , J. He , R. Ni , Q. Yang , Y. Zhang , and L. Luo , “Cerebrovascular Injuries Induce Lymphatic Invasion Into Brain Parenchyma to Guide Vascular Regeneration in Zebrafish,” Developmental Cell 49, no. 5 (2019): 697–710.e5. 31006646 10.1016/j.devcel.2019.03.022

[151] M. Prince , R. Bryce , E. Albanese , A. Wimo , W. Ribeiro , and C. P. Ferri , “The Global Prevalence of Dementia: A Systematic Review and Metaanalysis,” Alzheimer's & Dementia: The Journal of the Alzheimer's Association 9, no. 1 (2013): 63–75.e2. 10.1016/j.jalz.2012.11.007 23305823

[152] B. T. Kress , J. J. Iliff , M. Xia , et al., “Impairment of Paravascular Clearance Pathways in the Aging Brain,” Annals of Neurology 76, no. 6 (2014): 845–861. 25204284 10.1002/ana.24271 PMC4245362

[153] Y. Zhou , J. Cai , W. Zhang , et al., “Impairment of the Glymphatic Pathway and Putative Meningeal Lymphatic Vessels in the Aging Human,” Annals of Neurology 87, no. 3 (2020): 357–369. 31916277 10.1002/ana.25670

[154] Y. Zhou , W. Ran , Z. Luo , et al., “Impaired Peri‐Olfactory Cerebrospinal Fluid Clearance Is Associated With Ageing, Cognitive Decline and Dyssomnia,” EBioMedicine 86 (2022): 104381. 36442319 10.1016/j.ebiom.2022.104381 PMC9706530

[155] J. Rustenhoven , G. Pavlou , S. E. Storck , et al., “Age‐Related Alterations in Meningeal Immunity Drive Impaired CNS Lymphatic Drainage,” Journal of Experimental Medicine 220, no. 7 (2023): e20221929. 37027179 10.1084/jem.20221929 PMC10083715

[156] Z. Xu , N. Xiao , and Y. Chen , “Deletion of Aquaporin‐4 in APP/PS1 Mice Exacerbates Brain Aβ Accumulation and Memory Deficits,” Molecular Neurodegeneration 10 (2015): 58. 26526066 10.1186/s13024-015-0056-1 PMC4631089

[157] D. M. Zeppenfeld , M. Simon , J. D. Haswell , et al., “Association of Perivascular Localization of Aquaporin‐4 With Cognition and Alzheimer Disease in Aging Brains,” JAMA Neurology 74, no. 1 (2017): 91–99. 27893874 10.1001/jamaneurol.2016.4370

[158] D. M. Wilcock , M. P. Vitek , and C. A. Colton , “Vascular Amyloid Alters Astrocytic Water and Potassium Channels in Mouse Models and Humans With Alzheimer's Disease,” Neuroscience 159, no. 3 (2009): 1055–1069. 19356689 10.1016/j.neuroscience.2009.01.023 PMC2699894

[159] A. J. Thompson , S. E. Baranzini , J. Geurts , B. Hemmer , and O. Ciccarelli , “Multiple Sclerosis,” Lancet (London, England) 391, no. 10130 (2018): 1622–1636. 29576504 10.1016/S0140-6736(18)30481-1

[160] J. Rustenhoven and J. Kipnis , “Brain Borders at the Central Stage of Neuroimmunology,” Nature 612, no. 7940 (2022): 417–429. 36517712 10.1038/s41586-022-05474-7 PMC10205171

[161] M. van Zwam , R. Huizinga , M. J. Melief , et al., “Brain Antigens in Functionally Distinct Antigen‐Presenting Cell Populations in Cervical Lymph Nodes in MS and EAE,” Journal of Molecular Medicine (Berlin, Germany) 87, no. 3 (2009): 273–286. 19050840 10.1007/s00109-008-0421-4

[162] G. C. Furtado , M. C. Marcondes , J. A. Latkowski , J. Tsai , A. Wensky , and J. J. Lafaille , “Swift Entry of Myelin‐Specific T Lymphocytes Into the Central Nervous System in Spontaneous Autoimmune Encephalomyelitis,” Journal of Immunology (Baltimore, Md: 1950) 181, no. 7 (2008): 4648–4655. 18802067 10.4049/jimmunol.181.7.4648 PMC3973185

[163] M. van Zwam , R. Huizinga , N. Heijmans , et al., “Surgical Excision of CNS‐Draining Lymph Nodes Reduces Relapse Severity in Chronic‐Relapsing Experimental Autoimmune Encephalomyelitis,” Journal of Pathology 217, no. 4 (2009): 543–551. 19023878 10.1002/path.2476

[164] A. Cugurra , T. Mamuladze , J. Rustenhoven , et al., “Skull and Vertebral Bone Marrow Are Myeloid Cell Reservoirs for the Meninges and CNS Parenchyma,” Science (New York, NY) 373, no. 6553 (2021): eabf7844. 10.1126/science.abf7844 PMC8863069 34083447

[165] J. A. Mazzitelli , L. C. D. Smyth , K. A. Cross , et al., “Cerebrospinal Fluid Regulates Skull Bone Marrow Niches via Direct Access Through Dural Channels,” Nature Neuroscience 25, no. 5 (2022): 555–560. 35301477 10.1038/s41593-022-01029-1 PMC9081158

[166] M. Hsu , A. Rayasam , J. A. Kijak , et al., “Neuroinflammation‐Induced Lymphangiogenesis Near the Cribriform Plate Contributes to Drainage of CNS‐Derived Antigens and Immune Cells,” Nature Communications 10, no. 1 (2019): 229. 10.1038/s41467-018-08163-0 PMC6335416 30651548

[167] J. M. Park , Y. J. Shin , J. M. Cho , et al., “Upregulation of Vascular Endothelial Growth Factor Receptor‐3 in the Spinal Cord of Lewis Rats With Experimental Autoimmune Encephalomyelitis,” Journal of Histochemistry and Cytochemistry: Official Journal of the Histochemistry Society 61, no. 1 (2013): 31–44. 22983493 10.1369/0022155412462975 PMC3534318

[168] S. Schwager and M. Detmar , “Inflammation and Lymphatic Function,” Frontiers in Immunology 10 (2019): 308. 30863410 10.3389/fimmu.2019.00308 PMC6399417

[169] X. Hu , Q. Deng , L. Ma , et al., “Meningeal Lymphatic Vessels Regulate Brain Tumor Drainage and Immunity,” Cell Research 30, no. 3 (2020): 229–243. 32094452 10.1038/s41422-020-0287-8 PMC7054407

[170] E. Song , T. Mao , H. Dong , et al., “VEGF‐C‐Driven Lymphatic Drainage Enables Immunosurveillance of Brain Tumours,” Nature 577, no. 7792 (2020): 689–694. 31942068 10.1038/s41586-019-1912-x PMC7100608

[171] G. Oliver , J. Kipnis , G. J. Randolph , and N. L. Harvey , “The Lymphatic Vasculature in the 21(st) Century: Novel Functional Roles in Homeostasis and Disease,” Cell 182, no. 2 (2020): 270–296. 32707093 10.1016/j.cell.2020.06.039 PMC7392116

[172] A. Soumbasis , A. Ueno , D. Elliott , et al., “The Glymphatic System and Glioblastoma,” Brain: A Journal of Neurology (2025): awaf449. 41310975 10.1093/brain/awaf449 PMC13140616

[173] N. Mikhailov , A. Virenque , K. Koroleva , et al., “The Role of the Meningeal Lymphatic System in Local Meningeal Inflammation and Trigeminal Nociception,” Scientific Reports 12, no. 1 (2022): 8804. 35614095 10.1038/s41598-022-12540-7 PMC9133044

[174] M. A. Kovacs , M. N. Cowan , I. W. Babcock , et al., “Meningeal Lymphatic Drainage Promotes T Cell Responses Against Toxoplasma Gondii but Is Dispensable for Parasite Control in the Brain,” eLife 11 (2022): e80775. 36541708 10.7554/eLife.80775 PMC9812409

[175] X. Li , L. Qi , D. Yang , et al., “Meningeal Lymphatic Vessels Mediate Neurotropic Viral Drainage From the Central Nervous System,” Nature Neuroscience 25, no. 5 (2022): 577–587. 35524140 10.1038/s41593-022-01063-z

[176] E. Gousopoulos , S. T. Proulx , J. Scholl , M. Uecker , and M. Detmar , “Prominent Lymphatic Vessel Hyperplasia With Progressive Dysfunction and Distinct Immune Cell Infiltration in Lymphedema,” American Journal of Pathology 186, no. 8 (2016): 2193–2203. 27315777 10.1016/j.ajpath.2016.04.006

[177] M. Wang , C. Yan , X. Li , et al., “Non‐Invasive Modulation of Meningeal Lymphatics Ameliorates Ageing and Alzheimer's Disease‐Associated Pathology and Cognition in Mice,” Nature Communications 15, no. 1 (2024): 1453. 10.1038/s41467-024-45656-7 PMC10873306 38365740

[178] W. Zou , T. Pu , W. Feng , et al., “Blocking Meningeal Lymphatic Drainage Aggravates Parkinson's Disease‐Like Pathology in Mice Overexpressing Mutated α‐Synuclein,” Translational Neurodegeneration 8 (2019): 7. 30867902 10.1186/s40035-019-0147-y PMC6396507

[179] X. Lu , S. Bai , L. Feng , et al., “Cranial Bone Maneuver Ameliorates Alzheimer's Disease Pathology via Enhancing Meningeal Lymphatic Drainage Function,” Alzheimer's & Dementia: The Journal of the Alzheimer's Association 21, no. 2 (2025): e14518. 10.1002/alz.14518 PMC11848205 39887820

[180] X. F. He , D. X. Liu , Q. Zhang , et al., “Voluntary Exercise Promotes Glymphatic Clearance of Amyloid Beta and Reduces the Activation of Astrocytes and Microglia in Aged Mice,” Frontiers in Molecular Neuroscience 10 (2017): 144. 28579942 10.3389/fnmol.2017.00144 PMC5437122

[181] Y. Liu , P. P. Hu , S. Zhai , et al., “Aquaporin 4 Deficiency Eliminates the Beneficial Effects of Voluntary Exercise in a Mouse Model of Alzheimer's Disease,” Neural Regeneration Research 17, no. 9 (2022): 2079–2088. 35142700 10.4103/1673-5374.335169 PMC8848602

[182] L. S. B. Boisserand , L. H. Geraldo , J. Bouchart , et al., “VEGF‐C Prophylaxis Favors Lymphatic Drainage and Modulates Neuroinflammation in a Stroke Model,” Journal of Experimental Medicine 221, no. 4 (2024): e20221983. 38442272 10.1084/jem.20221983 PMC10913814

[183] O. Semyachkina‐Glushkovskaya , A. Abdurashitov , A. Dubrovsky , et al., “Photobiomodulation of Lymphatic Drainage and Clearance: Perspective Strategy for Augmentation of Meningeal Lymphatic Functions,” Biomedical Optics Express 11, no. 2 (2020): 725–734. 32206394 10.1364/BOE.383390 PMC7041454

[184] H. Ma , Y. Du , D. Xie , Z. Z. Wei , Y. Pan , and Y. Zhang , “Recent Advances in Light Energy Biotherapeutic Strategies With Photobiomodulation on Central Nervous System Disorders,” Brain Research 1822 (2024): 148615. 37783261 10.1016/j.brainres.2023.148615

[185] D. Nizamutdinov , C. Ezeudu , E. Wu , J. H. Huang , and S. S. Yi , “Transcranial Near‐Infrared Light in Treatment of Neurodegenerative Diseases,” Frontiers in Pharmacology 13 (2022): 965788. 36034819 10.3389/fphar.2022.965788 PMC9400541

[186] S. Bai , X. Lu , Q. Pan , et al., “Cranial Bone Transport Promotes Angiogenesis, Neurogenesis, and Modulates Meningeal Lymphatic Function in Middle Cerebral Artery Occlusion Rats,” Stroke 53, no. 4 (2022): 1373–1385. 35135326 10.1161/STROKEAHA.121.037912

[187] N. P. Nelson‐Maney , L. Bálint , and A. L. Beeson , “Meningeal Lymphatic CGRP Signaling Governs Pain via Cerebrospinal Fluid Efflux and Neuroinflammation in Migraine Models,” Journal of Clinical Investigation 134, no. 15 (2024): e175616. 38743922 10.1172/JCI175616 PMC11290972

[188] J. Chen , X. Li , R. Ni , et al., “Acute Brain Vascular Regeneration Occurs via Lymphatic Transdifferentiation,” Developmental Cell 56, no. 22 (2021): 3115–3127.e6. 34562378 10.1016/j.devcel.2021.09.005

[189] K. Kim , D. Abramishvili , S. Du , et al., “Meningeal Lymphatics‐Microglia Axis Regulates Synaptic Physiology,” Cell 188, no. 10 (2025): 2705–2719.e23. 40120575 10.1016/j.cell.2025.02.022 PMC12086007

[190] F. Salehpour , J. Mahmoudi , F. Kamari , S. Sadigh‐Eteghad , S. H. Rasta , and M. R. Hamblin , “Brain Photobiomodulation Therapy: A Narrative Review,” Molecular Neurobiology 55, no. 8 (2018): 6601–6636. 29327206 10.1007/s12035-017-0852-4 PMC6041198

[191] Y. Li , Y. Du , F. Ye , et al., “Photostimulation of Skull Bone Marrow Modulates Neuroimmunity in Sepsis‐Associated Encephalopathy via the Skull Bone Marrow‐Dura Mater‐Brain Axis,” Journal of Neuroinflammation 22, no. 1 (2025): 278. 41291851 10.1186/s12974-025-03617-x PMC12649072

[192] L. Tao , Q. Liu , F. Zhang , et al., “Microglia Modulation With 1070‐nm Light Attenuates Aβ Burden and Cognitive Impairment in Alzheimer's Disease Mouse Model,” Light, Science & Applications 10, no. 1 (2021): 179. 10.1038/s41377-021-00617-3 PMC8423759 34493703

[193] M. R. Hamblin , “Photobiomodulation for Traumatic Brain Injury and Stroke,” Journal of Neuroscience Research 96, no. 4 (2018): 731–743. 29131369 10.1002/jnr.24190 PMC5803455

[194] B. Xiao , “Levering Mechanically Activated Piezo Channels for Potential Pharmacological Intervention,” Annual Review of Pharmacology and Toxicology 60 (2020): 195–218. 10.1146/annurev-pharmtox-010919-023703 31454291

[195] J. Hu , Q. Chen , H. Zhu , et al., “Microglial Piezo1 Senses Aβ Fibril Stiffness to Restrict Alzheimer's Disease,” Neuron 111, no. 1 (2023): 15–29.e8. 36368316 10.1016/j.neuron.2022.10.021

[196] S. Chi , Y. Cui , H. Wang , et al., “Astrocytic Piezo1‐Mediated Mechanotransduction Determines Adult Neurogenesis and Cognitive Functions,” Neuron 110, no. 18 (2022): 2984–2999.e8. 35963237 10.1016/j.neuron.2022.07.010

[197] D. Choi , E. Park , E. Jung , et al., “Piezo1 Incorporates Mechanical Force Signals Into the Genetic Program That Governs Lymphatic Valve Development and Maintenance,” JCI Insight 4, no. 5 (2019): e125068. 30676326 10.1172/jci.insight.125068 PMC6483520

[198] D. Choi , E. Park , R. P. Yu , et al., “Piezo1‐Regulated Mechanotransduction Controls Flow‐Activated Lymphatic Expansion,” Circulation Research 131, no. 2 (2022): e2–e21. 35701867 10.1161/CIRCRESAHA.121.320565 PMC9308715

[199] S. Martin‐Almedina , S. Mansour , and P. Ostergaard , “Human Phenotypes Caused by PIEZO1 Mutations; One Gene, Two Overlapping Phenotypes?,” Journal of Physiology 596, no. 6 (2018): 985–992. 29331020 10.1113/JP275718 PMC5851881

[200] D. Choi , E. Park , J. Choi , et al., “Piezo1 Regulates Meningeal Lymphatic Vessel Drainage and Alleviates Excessive CSF Accumulation,” Nature Neuroscience 27, no. 5 (2024): 913–926. 38528202 10.1038/s41593-024-01604-8 PMC11088999

[201] M. J. Matrongolo , P. S. Ang , J. Wu , et al., “Piezo1 Agonist Restores Meningeal Lymphatic Vessels, Drainage, and Brain‐CSF Perfusion in Craniosynostosis and Aged Mice,” Journal of Clinical Investigation 134, no. 4 (2023): e171468. 37917195 10.1172/JCI171468 PMC10866656

[202] Y. H. Choi , M. Hsu , C. Laaker , et al., “Dual Role of Vascular Endothelial Growth Factor‐C in Post‐Stroke Recovery,” Journal of Experimental Medicine 222, no. 2 (2025): e20231816. 39665829 10.1084/jem.20231816 PMC11636551

[203] J. Y. Chen , D. W. Zhao , Y. Yin , et al., “Deep Cervical Lymphovenous Anastomosis (LVA) for Alzheimer's Disease: Microsurgical Procedure in a Prospective Cohort Study,” International Journal of Surgery (London, England) 111, no. 7 (2025): 4211–4221. 40391969 10.1097/JS9.0000000000002490

[204] Q. Xie , A. Louveau , S. Pandey , W. Zeng , and W. F. Chen , “Rewiring the Brain: The Next Frontier in Supermicrosurgery,” Plastic and Reconstructive Surgery 153, no. 2 (2024): 494e–495e. 37467388 10.1097/PRS.0000000000010933

[205] A. W. Wong , N. H. S. Sim , C. B. Thng , D. P. Y. Sim , Z. L. Low , and A. S. C. Foo , “The Potential Connection Between the Brain and Neck: Exploring the Meningeal‐Cervical Lymphatic Pathway in Primate Models,” Plastic and Reconstructive Surgery (2025). 10.1097/PRS.0000000000012627 41253126

